# Preparation of solid dispersion systems for enhanced dissolution of poorly water soluble diacerein: *In*-*vitro* evaluation, optimization and physiologically based pharmacokinetic modeling

**DOI:** 10.1371/journal.pone.0245482

**Published:** 2021-01-20

**Authors:** Shahinaze A. Fouad, Fady A. Malaak, Mohamed A. El-Nabarawi, Khalid Abu Zeid, Amira M. Ghoneim

**Affiliations:** 1 Department of Pharmaceutics, Faculty of Pharmacy, Ahram Canadian University, 6^th^ of October City, Giza, Egypt; 2 Department of Pharmaceutics and Industrial Pharmacy, Faculty of Pharmacy, Cairo University, Cairo, Egypt; 3 Department of Pharmaceutics and Pharmaceutical Technology, Faculty of Pharmaceutical Sciences and Pharmaceutical Industries, Future University in Egypt, Cairo, Egypt; ISF College of Pharmacy, Moga, Punjab, India, INDIA

## Abstract

Diacerein (DCN), a BCS II compound, suffers from poor aqueous solubility and limited bioavailability. Solid dispersion systems (SD) of DCN were prepared by solvent evaporation, using hydrophilic polymers. *In*-*vitro* dissolution studies were performed and dissolution parameters were evaluated. I-Optimal factorial design was employed to study the effect of formulation variables (drug:polymer ratio and polymer type) on the measured responses including; drug content (DC) (%), dissolution efficiency at 15 min (DE _(15 min)_%) and 60 min (DE _(60 min)_%) and mean dissolution time (MDT) (min). The optimized SD was selected, prepared and evaluated, allowing 10.83 and 3.42 fold increase in DE _(15 min)_%, DE _(60 min)_%, respectively and 6.07 decrease in MDT, compared to plain drug. DSC, XRD analysis and SEM micrographs confirmed complete amorphization of DCN within the optimized SD. Physiologically based pharmacokinetic (PBPK) modeling was employed to predict PK parameters of DCN in middle aged healthy adults and geriatrics. Simcyp^®^ software established *in*-*vivo* plasma concentration time curves of the optimized SD, compared to plain DCN. Relative bioavailability of the optimized SD compared to plain drug was 229.52% and 262.02% in healthy adults and geriatrics, respectively. Our study reports the utility of PBPK modeling for formulation development of BCS II APIs, via predicting their oral bio-performance.

## 1. Introduction

**Fig 1:**
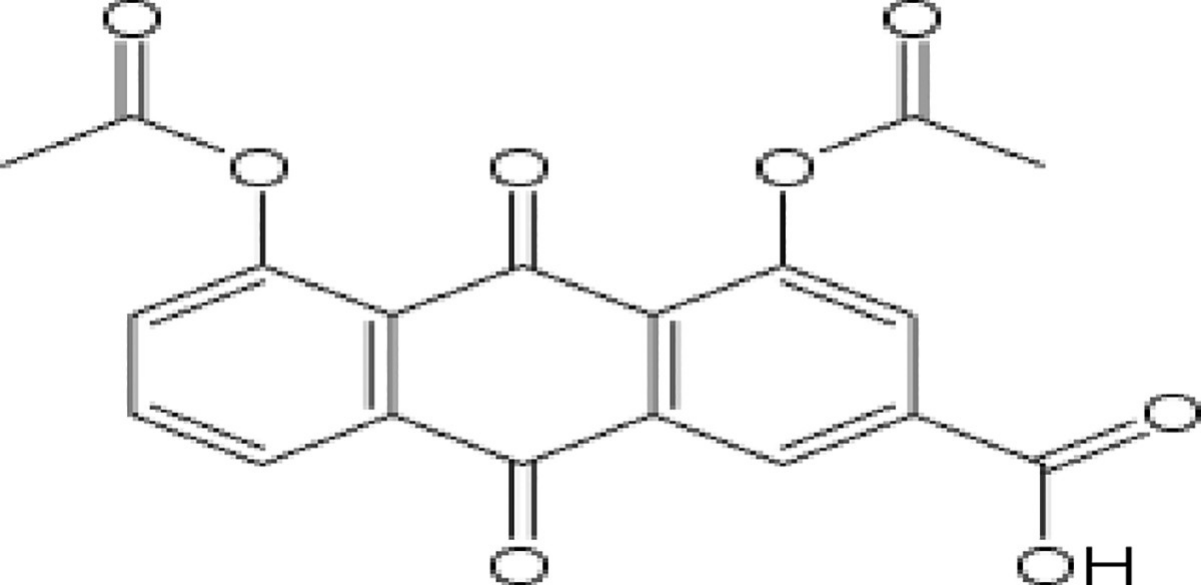
Chemical structure of DCN [1].

Diacerein (DCN) is chemically known as 4,5—diacetyloxy—9,10 dihydro-9, 10-dioxo-2-anthracenecarboxylic acid having the chemical structure shown in “Fig 1” [1]. It is an analgesic and anti-inflammatory drug used for treatment of osteoarthritis (OA) [2]. OA is a degenerative joint disorder that affects middle aged adults and geriatrics. It is characterized by progressive breaking down of cartilage in the affected joints, causing joint pain, swelling and stiffness [3]. Non-steroidal anti-inflammatory drugs (NSAIDs) are the first line of treatment for disease-associated pain [4]. The potential benefits of DCN exist in being a successful alternative to NSAIDs with the advantage of excluding gastric side effects [5]. In addition, it acquires disease modifying properties [6] (pro-anabolic/chondro-protective effects), which are highly beneficial in this chondro-destructive disease [7]. Yet, DCN belongs to Biopharmaceutics Classification System, class II (BCS II) active pharmaceutical ingredients (APIs). It is poorly soluble, with reported aqueous solubility of 3.197 mg/mL [8]. As a result, it suffers from poor dissolution properties, reduced oral absorption [5] and consequently, lowered bioavailability equal to 35–56% [9].

Various techniques have been studied for enhancing solubility and dissolution rates of poorly soluble drugs, for achieving improved oral absorption and bioavailability such as; micronization [10], complexation [11], co-precipitation/co-grinding [12], spray drying [13], freeze-drying [14] and solid dispersion formation [15]. Because DCN exhibits an insoluble, crystalline nature, intentional solid state modification (from crystalline to amorphous form) can be promising in enhancing its solubility, dissolution properties and in return its bioavailability. In our study, solid dispersions (SD) of DCN are aimed to be prepared by solvent evaporation technique. This method involves incorporation of a hydrophobic drug into a hydrophilic polymer in one system. Polyvinyl pyrrolidones (PVPs) and polyethylene glycols (PEGs) are water soluble polymers constantly used in preparation of SD of poorly soluble APIs due to their favorable hydrophilic properties. Thus, the aim of the current study is to prepare SD of DCN using different molecular weights of PVPs and PEGs, at various “Drug:Polymer” (D:P) ratios aiming to enhance the *in*-*vitro* dissolution properties of DCN.

*In*-*vitro* dissolution results of oral formulations belonging to BCS II APIs can timidly predict their *in*-*vivo* pharmacokinetic (PK) performance. This is because their oral absorption is restricted by their *in*-*vivo* dissolution manner. Thus, establishment of an *in*-*vitro*/*in*-*vivo* correlation (IVIVC) for these drugs plays a crucial role in drug development. Such correlation combines the drug’s biochemical/physicochemical factors with the populations’ anatomical/physiological factors, together with the formulation properties of the drug’s product, in one simulating model. Physiologically based pharmacokinetic (PBPK) modeling is a mechanistic tool that is currently widely employed to predict PK properties of drug formulations in various populations. As reported by Parrott et al. [16], PBPK modeling is an integrated part in prediction of human PK, as well as preclinical research and development of new drugs. This is because it enables the prediction of plasma concentration time curves from *in*-*vitro* data, providing a useful source supporting decisions during the different phases of drug development [17]. It is a simulation top-down approach that involves estimation of model parameters via clinically reported PK data [18] where, successful simulation of *in*-*vivo* fate and accurate PK parameters of formulations can be achieved. Recently, PBPK modeling has received great attention for the prediction of systemic drug concentrations in healthy and special populations [19]. Also, prediction of inter-individual variabilities accompanied by absorption, distribution, metabolism and excretion (ADME) of administered drugs has been achieved by PBPK modeling [20, 21]. In addition, it can promote development of drug-disease model via incorporation of pathophysiological variabilities occurring within a certain disease [22]. Once this model is constructed and assessed, it can be efficiently extrapolated to other drugs and populations [23].

As a result, nowadays the number of publications and regulatory submissions utilizing and implementing PBPK modeling has greatly increased, being utilized and implemented as a successful predictive alternative to *in*-*vivo* experimental studies. These include many studies such as; a study by Lin et al. [24], where authors utilized the PBPK modeling to convert the *in*-*vitro* estrogen receptor assays to human equivalent doses, eliminating the combined risk assessment of endocrine effects of bisphenol A (BPA) and its analogues. In another study by Fabian et al. [25], they succeeded in using Physiologically Based Toxicokinetic (PBTK) rat model based on *in*-*vitro* and *in*-*silico* input data to replace *in*-*vivo* animal testing for toxicological hazard assessment of potential endocrine-disrupting compounds. Moreover, in another study by Ellison et al. [26], PBPK modeling approach, using *in*-*vitro* and *in*-*silico* inputs, was applied to develop human oral PBPK models for caffeine and diphenhydramine. In addition, Kovar et al. [27] utilized the PBPK modeling in successfully predicting the pharmacokinetics of buprenorphine in children, specifically in neonates, where conducting clinical trials within this population is really challenging. It was also reported by Rioux et al. [28] that application of PBPK modeling in drug development for pediatric cancers is relatively nascent where, the regulatory authorities with the United States Food and Drug administration (FDA) have recommended the application of PBPK modeling in the FDA Strategic Plan for accelerating the development of therapies for pediatric diseases.

To the best of our knowledge, literature lack published studies employing PBPK modeling to predict PK profiles of DCN, in middle aged adults and geriatrics. Since it is uneasy to collect PK data from geriatrics, in specific, due to comorbidities and coexisting drug therapies, simulation results in our study can be greatly helpful in clinical studies of prevailing diseases as OA. Therefore, as previously mentioned the overall aim of our present study is to enhance the poor dissolution properties of DCN, where, *in*-*vitro* dissolution parameters are thoroughly investigated compared to plain form of the drug and statistically analyzed in order to predict the optimized SD system of DCN. The obtained results are coherently enrolled to establish an IVIVC via implementing PBPK modeling in order to predict the oral bio-performance of this BCS II API and confirm the bioavailability enhancement of the optimized SD compared to plain DCN.

## 2. Materials and methods

### 2.1. Materials

Diacerein (DCN) was kindly supplied by EVA Pharm Egypt. Polyethylene glycol (PEG) 4000, PEG 8000, Polyvinylpyrrolidone (PVP) K25 and PVP K90 were supplied from Fluka AG Buchs SG, Switzerland. Disodium hydrogen orthophosphate, potassium di-hydrogen orthophosphate were supplied from El-Nasr Company for pharmaceuticals, Cairo, Egypt. Distilled water was used throughout the study. All other chemicals were reagent grade and used as received.

### 2.2. Compatibility studies of hydrophilic polymers for dissolution enhancement of DCN

#### 2.2.1. Differential Scanning Calorimetry (DSC) studies

For rapid screening of drug/polymer interactions, DSC studies were performed [29]. Samples (5 mg) of plain DCN, hydrophilic polymers and their physical mixtures (PM) (in the ratio 1:1 w/w) were sealed in aluminum pans, analyzed using Shimadzu DSC-50 (Kyoto, Japan) and heated in nitrogen atmosphere where, the heating rate was constant (10°C/min) within 20–300°C. DSC curves of samples under study were determined under same experimental conditions.

#### 2.2.2. Fourier transform infra-red spectroscopy (FTIR)

DCN, hydrophilic polymers and their PM (in the ratio 1:1 w/w) were analyzed using Shimadzu FTIR-435, Kyoto, Japan in scanning range 400 to 4000 cm^-1^. Each sample (5 mg) was mixed with 100 mg potassium bromide and compressed into discs under pressure.

### 2.3. Preparation of solid dispersion systems (SD) of DCN

SD were prepared via the solvent evaporation method using Rotary evaporator (Heidolph, Laborota 4000 efficient, USA). The procedure was performed by constant mixing of the drug and hydrophilic polymers at 60°C in an appropriate solvent; mixture of ethyl alcohol and phosphate buffer pH 6.8 in a ratio 1:1 v/v, in order to ensure optimum solubility of both drug and the polymers. The resulting systems were cooled to room temperature and a solid mass of each system was obtained. Each mass was crushed and passed through a sieve (mesh size 100) to get uniform sized particles. The prepared SD were kept in tightly closed containers over calcium chloride at room temperature until further analysis.

### 2.4. *In*-*vitro* dissolution studies

Dissolution profiles of DCN from the prepared systems compared to plain drug were determined using USP Rotating Paddle Apparatus II (Pharma Test Dissolution Tester, Germany) at 37°C ± 0.5°C and 100 revolutions per minute (r.p.m), by the dispersed powder method [30]. Plain drug (50 mg) and an amount of each of the prepared systems (equivalent to 50 mg DCN) were accurately weighed and placed in a dissolution flask containing 900 mL phosphate buffer (pH 6.8). Five mL samples were withdrawn at specified time intervals and replaced with an equal volume of fresh dissolution medium to maintain a constant total volume. The collected samples were filtered using a syringe filter (having 0.45 μm pore size), suitably diluted with phosphate buffer (pH 6.8) and assayed spectrophotometrically at 258 nm. *In*-*vitro* dissolution tests were carried out in triplicate (n = 3 ± S.D.). Similarity factor (*f*2), a mathematical approach that is model independent was calculated in order to compare dissolution profiles of plain DCN with those of the prepared SD. *f*2 values < 50 suggest significant difference between dissolution profiles under comparison. The following equation (Eq (1)) was used for *f*2 calculation [31]:

$$f_{2}=50\;\log\left\{{\left[1+\frac{1}{n}\sum_{t=1}^{n}{\left(R_{t}-T_{t}\right)}^{2}\right]}^{0.5}x\;100\right\}$$ (1)

### 2.5. Evaluation of the prepared SD

#### 2.5.1. Drug content (DC) determination

DC was determined by dissolving an accurately weighed amount of each system (equivalent to 50 mg DCN) in 100 mL phosphate buffer (pH 6.8). The solution was filtered through 0.45 μm membrane filter and absorbance was measured spectrophotometrically by UV Spectrophotometer (Shimadzu, UV-2401 PC, Australia) at the predetermined λmax (258 nm) after appropriate dilution. All experiments were performed in triplicate (n = 3 ± S.D.).

#### 2.5.2. Determination of dissolution parameters

In order to compare the effect of different D:P ratios, as well as different types of polymers within the prepared systems, other model independent approaches were employed. These include; dissolution efficiency percent (DE %) and the mean dissolution time (MDT). In our study, values of DE % at 15 min (DE _(15 min)_ %) and 60 min (DE _(60 min)_ %) for plain drug, as well as each system was calculated as percent ratio of the area under dissolution profiles curve (up to the time (t) where, t = 15 and 60 min, respectively), to the area of the rectangle attaining 100% dissolution at same time. DE % was calculated according to the following equation (Eq (2)) [32]:

$$Dissolution\;efficiency\;(\%)=\frac{\int_{t1}^{t2}ydt}{y_{100}(t_{2}-t_{1})}\times 100$$ (2)

The rate of drug dissolved was expressed by the mean dissolution time (MDT) and was determined for each system using the following equation (Eq (3)):

$$MDT=\frac{\sum_{j=1}^{n}t_{j}^{*}\Delta M_{j}}{\sum_{j=1}^{n}\Delta M_{j}}$$ (3)

Where, (j) is the number of the sample, (n) is the number of samples under dissolution, (t*_j_) is the midpoint time between t and t_(j-1)_ and (ΔM_j_) is the additional amount of drug dissolved between t and t_(j-1)_ [33]. Model independent approaches were computed using the DD-Solver program, an add-in program for Microsoft excel, employed to compare dissolution profiles of different formulations [34].

#### 2.5.3. Kinetic analysis of the *in*-*vitro* dissolution profiles data

In order to understand the mode of DCN dissolution from the prepared systems, as well as the plain drug, kinetic analysis was determined by finding the best fit of dissolution profiles data (percent of drug dissolved versus time) to distinguished models; zero order, first order and Higuchi diffusion model as follows [35]:

Zero order equation: *C* = *C*_0_−*K*_0_*t*
First order equation: *log C* = *log C*_0_−*Kt*/2.303
Higuchi diffusion model equation: *Q* = *K*×*t*^1/2^

Preference of a certain mechanism was based on the correlation coefficient (R^2^) for dissolution parameters under study. Highest R^2^ indicates the preferable model [36].

### 2.6. Formulation optimization

I-Optimal factorial design was employed to evaluate the effects of formulation variables on the measured responses. The studied independent formulation variables were the drug:polymer (D:P) ratio (A) and the polymer type (B). The chosen responses were drug content (Y_1_: DC), dissolution efficiency % at 15 min (Y_2_: DE _(15 min)_ %), dissolution efficiency % at 60 min (Y_3_: DE _(60 min)_ %) and the mean dissolution time (Y_4_: MDT) (Table 1). Measured responses of the prepared systems are listed in Table 2. All responses were treated by Design Expert^®^ software (Version 10.0.3, Stat-Ease Inc. Minneapolis, MN, USA). Each response was analyzed individually and fitted to suitable statistical models including linear, two-factor interaction (2FI) and quadratic models using linear regression. The best fitted mathematical model was chosen based on comparisons of several statistical parameters including multiple correlation coefficient (R^2^), adjusted multiple correlation coefficient (adjusted R^2^), predicted multiple correlation coefficient (predicted R^2^) and the predicted residual sum of squares (PRESS). For the selected model, Design expert software^®^ suggests the highest adjusted R^2^, the highest predicted R^2^, as well as the lowest PRESS [37]. Analysis of variance (ANOVA) was implemented to determine model significance at α = 0.05 significance level, where the model (linear, cubic or quadratic) is considered significant when the p value ≤ 0.05. In addition, line plots were executed to point out the significant impact of independent variables on the measured responses, as well as to select the optimum levels of each variable. The optimized system was selected based on desirability calculations in order to attain maximum DC, DE _(15 min)_ % and DE _(60 min)_ %, as well as minimum MDT. The optimized system was then prepared and evaluated in triplicate to ensure that the measured responses given by software is valid then, further investigations took place.

**Table 1:** I-Optimal factorial design used to optimize solid dispersion systems of DCN.

Independent variables				
A: Drug:Polymer ratio	Levels			
	1:1	1:2	1:4	
B: Polymer type	PVP K25	PVP K90	PEG 4000	PEG 8000
Dependent variables	Constraints			
Y_1_: DC (%)	Maximize			
Y_2_: DE _(15 min)_ %	Maximize			
Y_3_: DE _(60 min)_ %	Maximize			
Y_4_: MDT (min)	Minimize			

**Table 2:** Experimental runs, formulation variables, measured responses of the I-Optimal factorial design and similarity factor.

Runs	Formulation variables		Measured responses				Similarity factor (*f*2)
	D:P ratio (A)	Polymer type (B)	Y_1_: Drug Content (% ± SD)	Dissolution Parameters			
				Y_2_: DE_(15 min)_ %	Y_3_: DE_(60 min)_ %	Y_4_: MDT (min)	
SD1	1:1	PVP K-25	95.52 ± 1.81	35.23	75.66	11.56	15.88
SD2	1:1		93.98 ± 1.53	33.31	73.12	12.55	17.61
SD3	1:2		93.99 ± 1.03	52.71	71.66	13.78	14.52
SD4	1:2		89.76 ± 0.05	51.28	72.55	12.86	15.67
SD5	1:4		96.00 ± 0.95	28.57	76.97	10.88	20.20
SD6	1:1	PVP K-90	92.61 ± 1.14	31.79	63.13	19.10	21.98
SD7	1:1		89.45 ± 0.62	29.65	61.18	20.88	23.07
SD8	1:2		93.59 ± 1.47	13.69	40.03	28.47	42.10
SD9	1:2		96.20 ± 0.04	15.05	42.70	26.81	39.36
SD10	1:4		96.52 ± 0.71	36.98	67.33	15.99	17.10
SD11	1:1	PEG 4000	93.13 ± 0.08	39.29	54.82	14.11	25.14
SD12	1:2		96.71 ± 0.81	27.50	43.93	16.52	35.77
SD13	1:2		100.68 ± 1.28	27.37	41.95	18.16	36.74
SD14	1:4		98.89 ± 0.07	39.97	70.55	16.04	18.18
SD15	1:1	PEG 8000	98.76 ± 1.73	68.13	81.65	8.12	10.64
SD16	1:2		92.47 ± 1.11	46.57	77.08	10.88	14.81
SD17	1:2		91.30 ± 1.78	44.81	75.17	11.60	15.70
SD18	1:4		98.01 ± 0.07	78.35	95.92	4.55	6.36

### 2.7. Evaluation of the optimized SD

#### 2.7.1. DSC

Thermograms of plain DCN and the optimized SD were determined using the same method as previously mentioned under section (2.2.1).

#### 2.7.2. X-ray diffraction (XRD) study

XRD study was performed for crystallinity assessment. Experiments were performed in a Scintag Corp. X-ray diffractometer (USA) using Cu K-alpha radiator with a nickel filter. Voltage of 45 kV and current of 40 mA were used. Diffraction patterns of plain drug powder and the optimized SD were determined.

#### 2.7.3. Scanning electron microscopic (SEM) analysis

Surface morphology of plain drug powder and the optimized SD were examined employing JSM-6390 LV, JEOL scanning electron microscope, Tokyo, Japan. Powders were fixed on a brass stub using double sided adhesive tape, then made electrically conductive by coating in a vacuum chamber with a thin layer of gold for 30 s. SEM photographs were taken at an increasing voltage of 20 kV.

### 2.8. Physiologically based pharmacokinetic (PBPK) modeling

Simcyp^®^ simulator software (V17.1, Certara, Sheffield, UK) was employed to establish a PBPK model of DCN for middle aged adults and geriatrics. The applied model was “Advanced Dissolution, Absorption and Metabolism” (ADAM). This model was based on physicochemical properties of DCN and DCN PK parameters reported in literature (Table 3). It also incorporates formulation factors for simulation obtained from *in*-*vitro* dissolution data of the optimized SD, as well as plain DCN. Ten simulated trials were done on ten healthy volunteers (n = 100), under fasting conditions to estimate the PK parameters. The dose was set to 50 mg, given once with 150 mL water. PK parameters including; the maximum plasma concentration (C_max_, μg/mL), the time required to reach the C_max_ (T_max_, h) and the area under the plasma concentration time curve (AUC_0-24h_, μg/mL.h) were predicted and compared to the reported data from the clinical study performed by Nguyen et al. [1].

**Table 3:** Modeling parameters of DCN.

Parameters	Description	References
Molecular weight (g/mol)	368.29	[38]
pKa	3.37	[38]
Log P^a^	2.14	[38]
Type	Monotropic acid	[38]
B/P^b^	0.604	Predicted by Simcyp^®^ software
Fu^c^	0.010	[39]
V_ss_^d^ (L/Kg)	0.23	[39]
CL^e^ (L/h)	1.5	[39]

^a^Log P: oil-water partition coefficient

^b^B/P: Blood/plasma ratio

^c^Fu: Fraction of drug unbound in plasma

^d^V_ss_: apparent volume of distribution and

^e^CL: oral clearance.

## 3. Results and discussion

### 3.1. Compatibility studies of hydrophilic polymers for dissolution enhancement of DCN

#### 3.1.1. DSC studies

**Fig 2:**
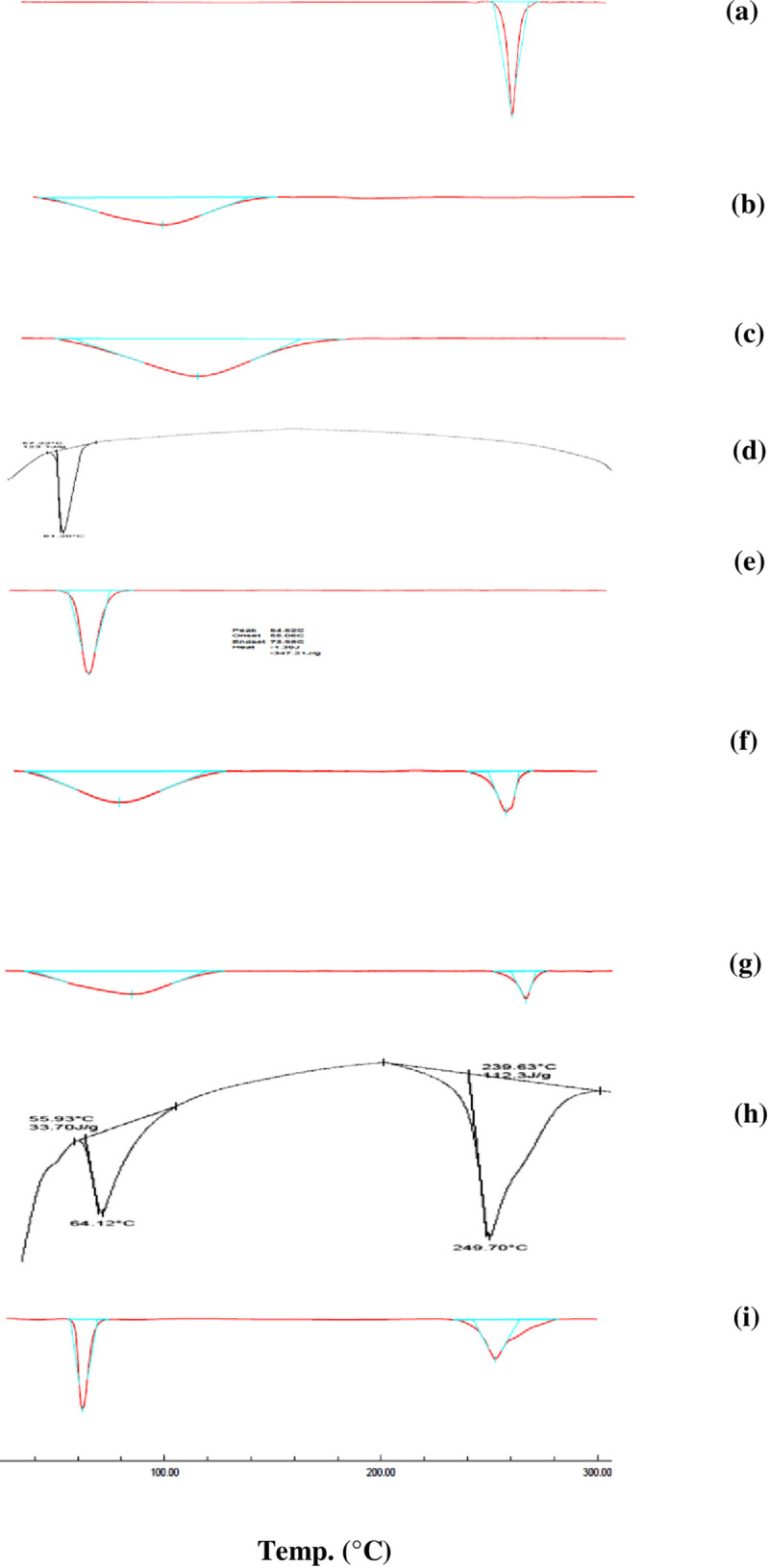
DSC compatability studies of plain drug (a), PVP K25 (b), PVP K90 (c), PEG 4000 (d), PEG 8000 (e), PM of drug and PVP K25 (f), PM of drug and PVP K90 (g), PM of drug and PEG 4000 (h) and PM of drug and PEG 8000 (i).

DSC thermograms of plain DCN, hydrophilic polymers and their PM are shown in “Fig 2A–2I”. “Fig 2A” showed that DCN is characterized by one sharp endothermic peak at 256.82°C, corresponding to its melting point at the crystalline state. DSC patterns of hydrophilic polymers are shown individually in “Fig 2B–2E”. DSC thermograms of PM are shown in “Fig 2F–2I” where two peaks are displayed, corresponding to both the drug and the hydrophilic polymer. The characteristic peak of the drug was preserved in all physical mixtures reflecting retained drug crystallinity, however its lower intensity was a result of dilution due to physical mixing.

#### 3.1.2. FTIR studies

**Fig 3:**
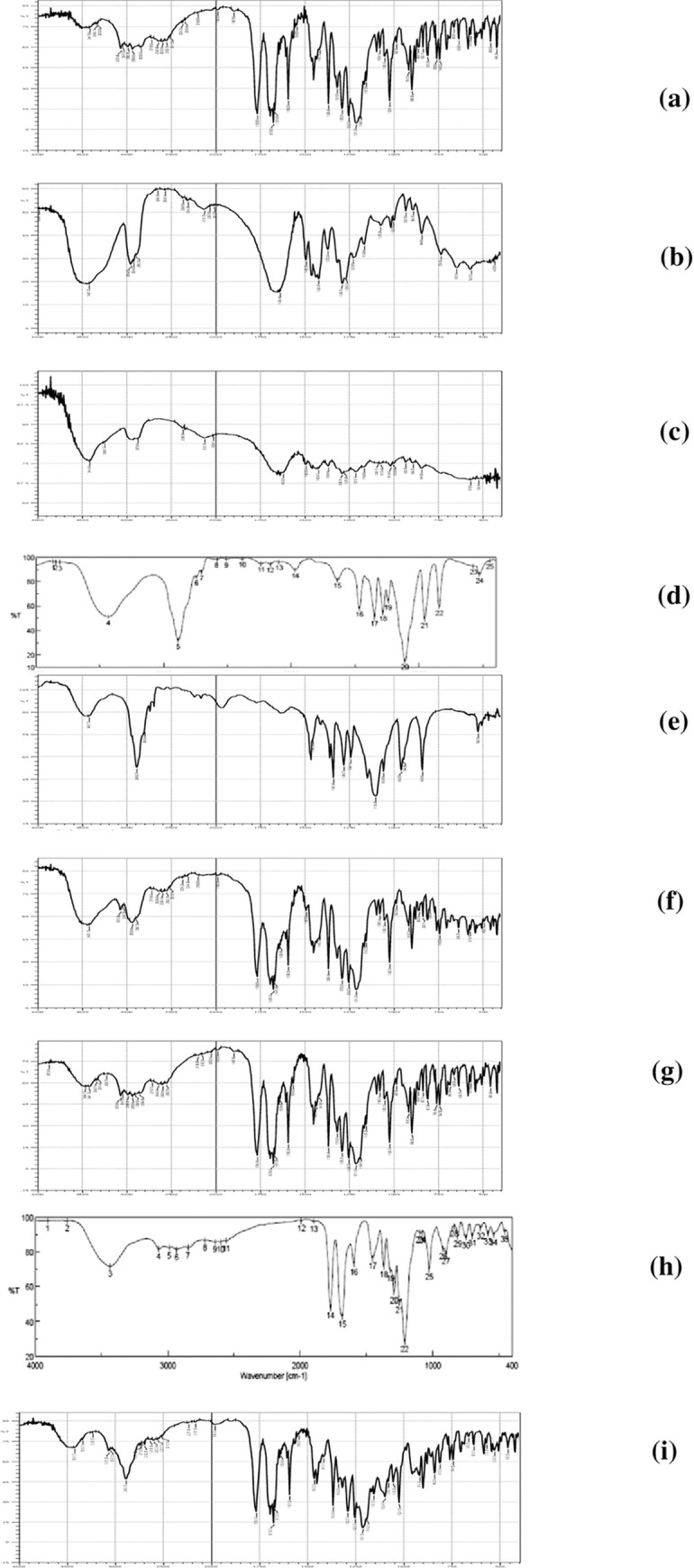
FTIR compatability studies of plain drug (a), PVP K25 (b), PVP K90 (c), PEG 4000 (d), PEG 8000 (e), PM of drug and PVP K25 (f), PM of drug and PVP K90 (g), PM of drug and PEG 4000 (h) and PM of drug and PEG 8000 (i).

FTIR spectra were determined to denote molecular drug changes resulting from drug-polymer interactions [40]. In our study, FTIR spectrum of pure DCN showed its characteristic absorption bands which appeared to be allocated at 3421 cm^-1^ (O-H, stretch, broad, COOH), 3066 cm^-1^ (C-H, stretch, aromatic), 2935 cm^-1^ (C-H, stretch, aliphatic, sym), 1770 cm^-1^ (C = O, stretch, ester), 1678 cm^-1^ (C = O, stretch, COOH), 1670 cm^-1^ (C = O, stretch, Ketone), 1593 cm^-1^ (C = C, stretch, aromatic) and 704 cm^-1^ (benzene) (Fig 3A). “Fig 3B–3E” showed FTIR spectra of hydrophilic polymers and “Fig 3F–3I” showed FTIR spectra of PM. Maintained drug bands within these spectra indicates lack of chemical interaction between DCN and hydrophilic polymers under study.

### 3.2. *In*-*vitro* dissolution studies

**Fig 4:**
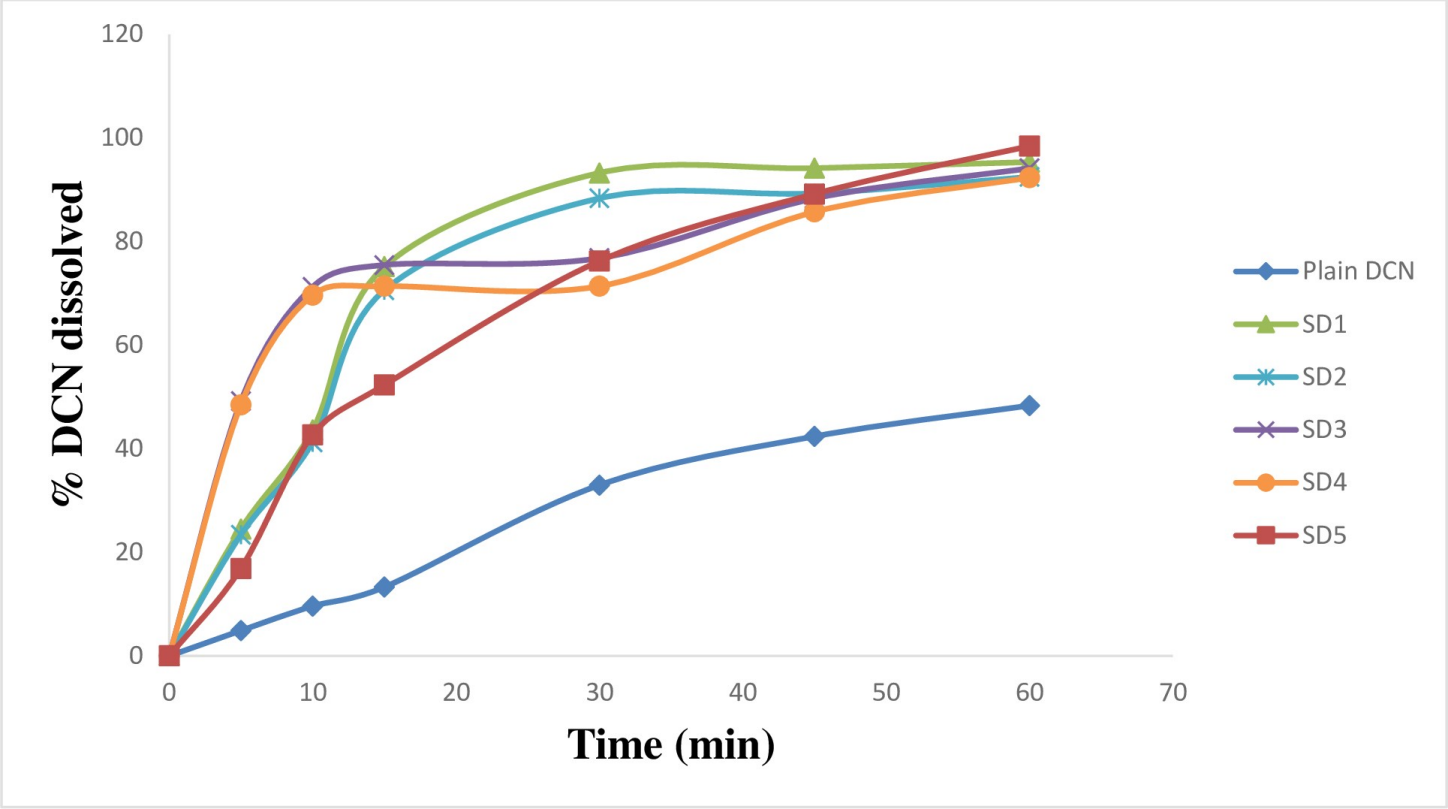
*In*-*vitro* dissolution profiles of DCN-SD containing different D:P ratios of PVP K25, compared to plain DCN.

**Fig 5:**
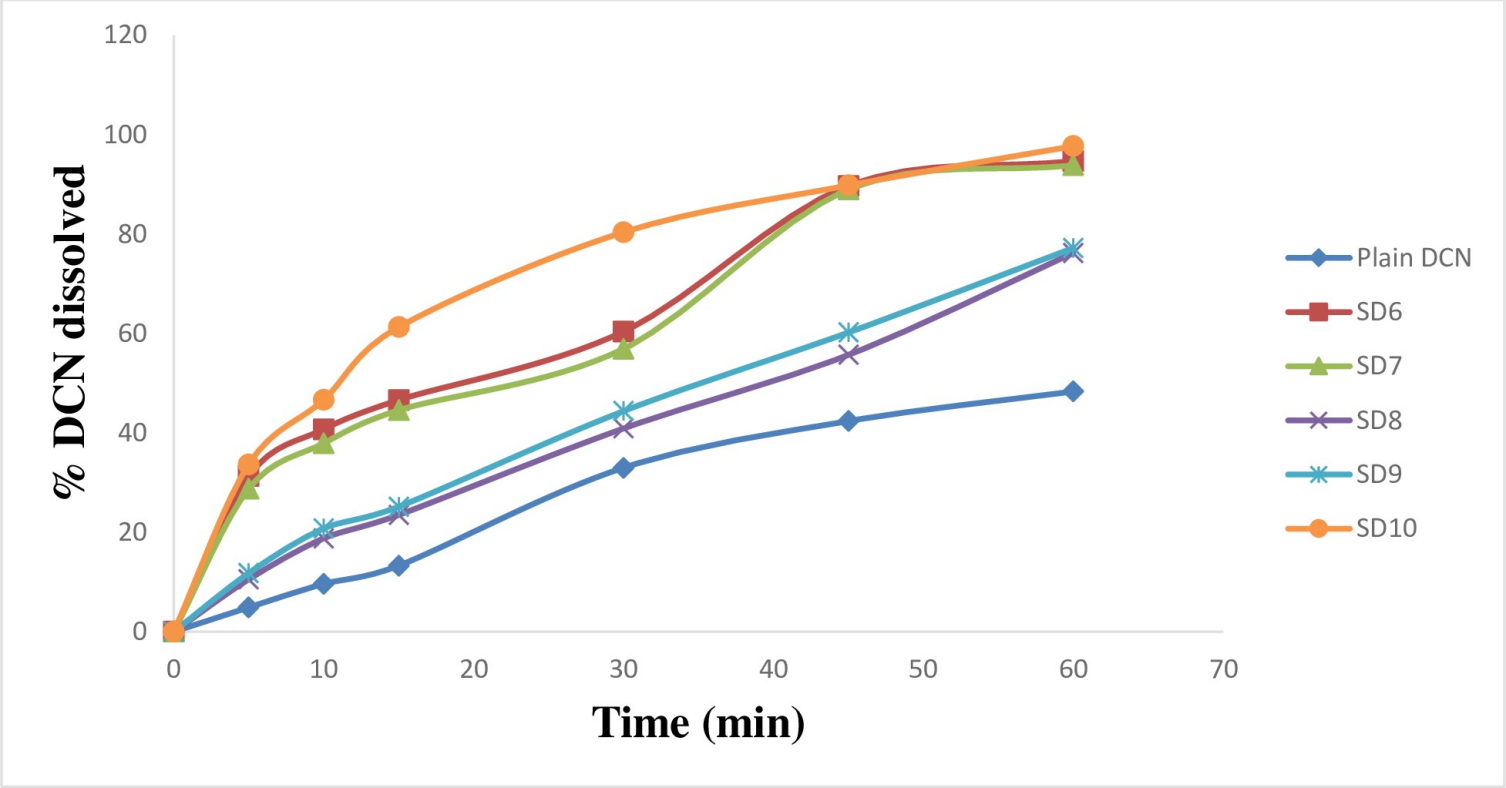
*In*-*vitro* dissolution profiles of DCN-SD containing different D:P ratios of PVP K90, compared to plain DCN.

**Fig 6:**
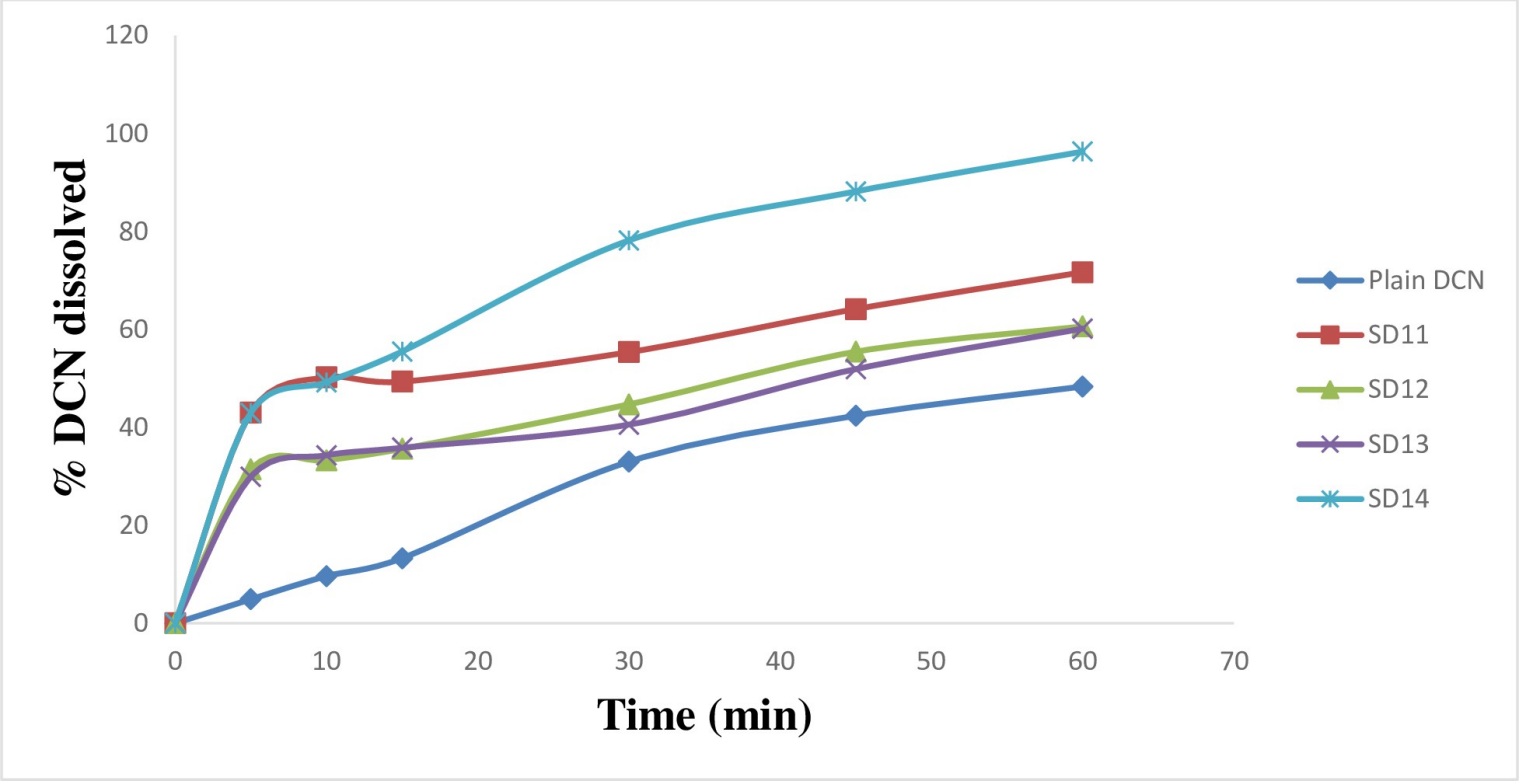
*In*-*vitro* dissolution profiles of DCN-SD containing different D:P ratios of PEG 4000, compared to plain DCN.

**Fig 7:**
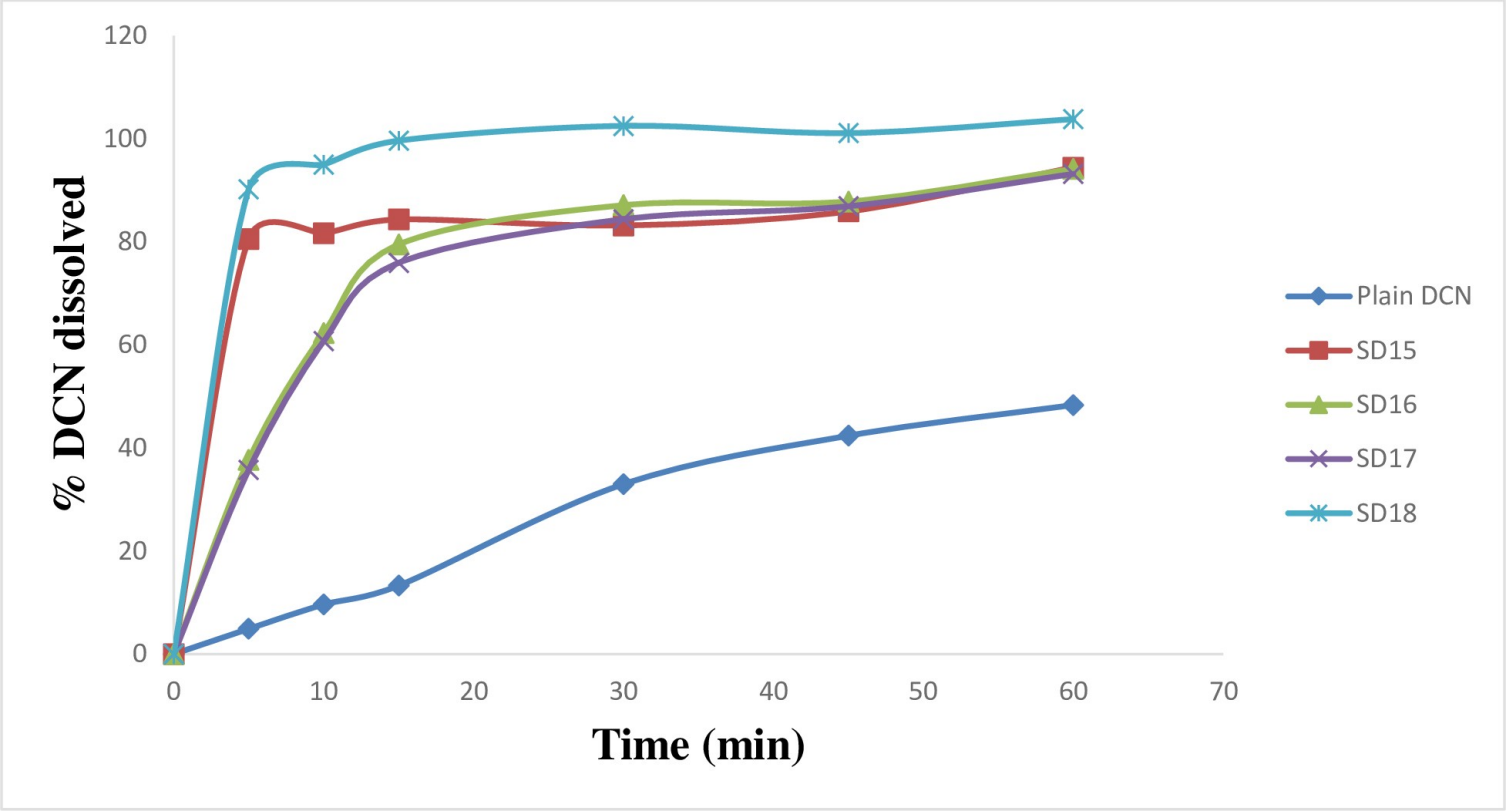
*In*-*vitro* dissolution profiles of DCN-SD containing different D:P ratios of PEG 8000, compared to plain DCN.

*In*-*vitro* dissolution of DCN was studied using phosphate buffer (pH 6.8) as dissolution medium. Enhanced dissolution rate of DCN was attained in all cases (Figs 4–7). Dissolution rate of the prepared SD significantly exceeded the plain drug (*f*2 values were found to be less than 50 for all systems) (Table 2). Plain drug showed significantly sluggish and incomplete dissolution (48.345 ± 2.01%) within the total dissolution time (one hour (h)). However, dissolution of all systems almost reached completion at the end of dissolution time. The maximum and minimum drug dissolved were recorded by SD18 (103.8 ± 0.06%) and SD8 (76.17 ± 1.02%), respectively after 60 minutes (min). Results showed that the highest dissolution was achieved from systems prepared at 1:4 (w/w) D:P ratio, whereas the least dissolution was obtained from systems prepared at 1:2 (w/w) D:P ratio. Accordingly, D:P ratios can be arranged in the following manner 1:4 > 1:1 > 1:2. Also, results revealed that PEG 8000 based SD systems displayed superior dissolution rates compared to other prepared systems. Improved dissolution rate of DCN within the prepared systems can be attributed to several factors including incorporation of strongly hydrophilic polymers to enhance drug’s wettability. Enhanced wetting properties of hydrophobic DCN resulted in localized enhancement of its solubility within the diffusion layer surrounding drug particles. Similar results were obtained by El-nawawy et al. [41] in their study on olmesartan solid dispersions. In addition, these hydrophilic polymers are considered as precipitation inhibitors, where they produce a shell of hydration around drug molecules, preventing their aggregation [42]. Moreover, they are able to form physically stable, soluble complexes with DCN via intermolecular hydrogen bonding. DCN contains one hydroxyl group which can form hydrogen bonds with carbonyl oxygen of the amide group in PVP molecules and ether oxygen in PEG molecules. Through hydrogen bond formation, polymers bind to the surface of drug particles preventing their nucleation and crystal growth. Sekizaki et al. [43] and Turhan et al. [44] confirmed hydrogen bond formation due to the presence of these functional groups in both polymers, respectively. It was also reported that stabilizing polymers as PVP acquire an antiplasticizing effect, providing decreased drug molecular mobility which inhibits drug nucleation/aggregation and hence contribute to drug amorphization [45, 46]. Finally, amorphous form of DCN requires low energy to be dissolved where, reduced particle size increased surface area of drug particles subjected to dissolution medium. All these factors could contribute to enhanced dissolution profiles of DCN within the prepared SD systems.

### 3.3. Statistical analysis of measured responses

#### 3.3.1. Drug content (DC %)

**Fig 8:**
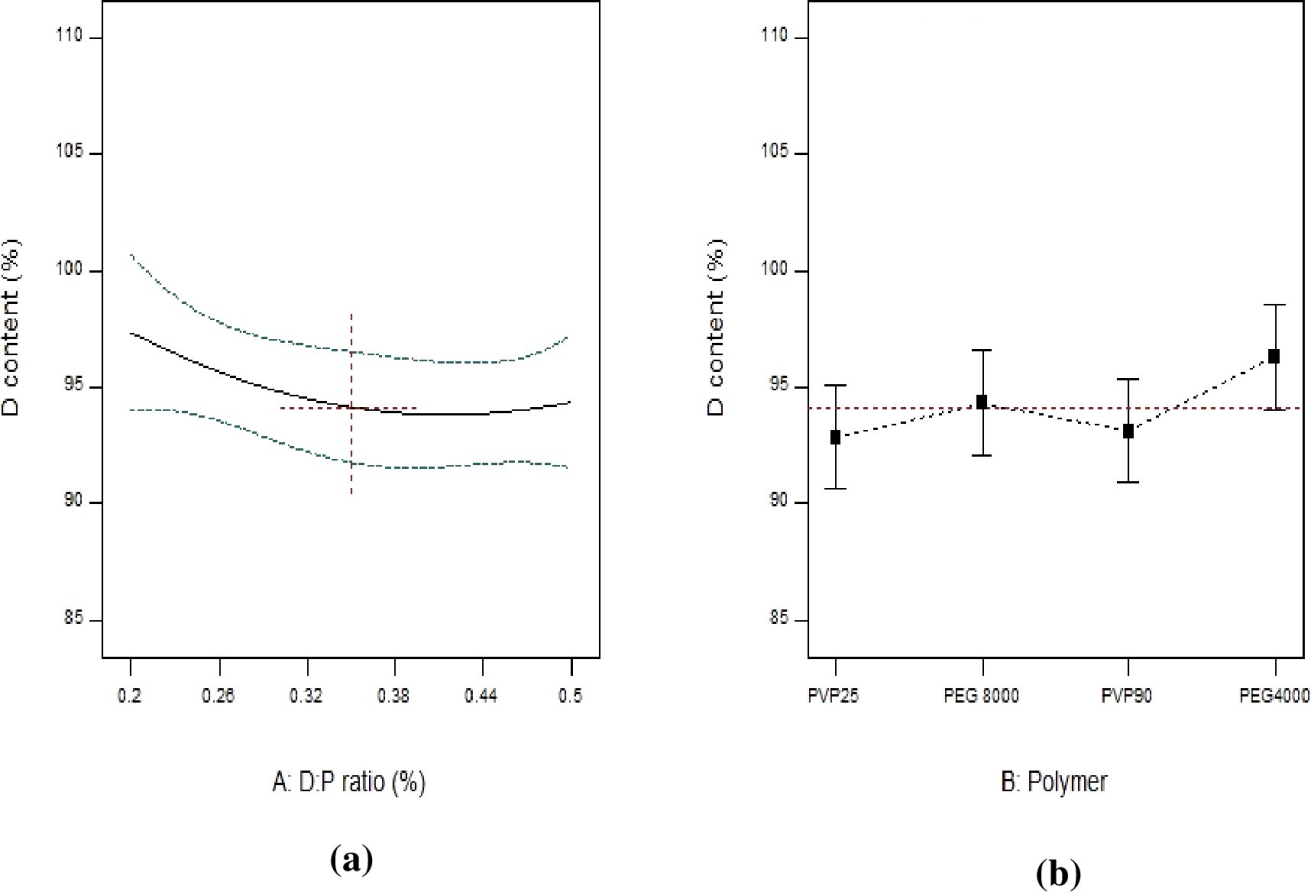
Line plots for the effect of formulation variables on DC %.

The mean percent of drug content ranged from 89.45 ± 0.62 to 100.68 ± 1.28 indicating its uniformity within the prepared SD. Results are listed in Table 2. “Fig 8A” shows the response line plot illustrating the significant effect of varying D:P ratios (A) on DC % within the prepared systems (Y_1_) (p = 0.0104). ANOVA results also revealed the significant effect of B on Y_1_ (p < 0.0001) (Fig 8B).

#### 3.3.2. DE _(15 min)_ %, DE _(60 min)_ % and MDT

**Fig 9:**
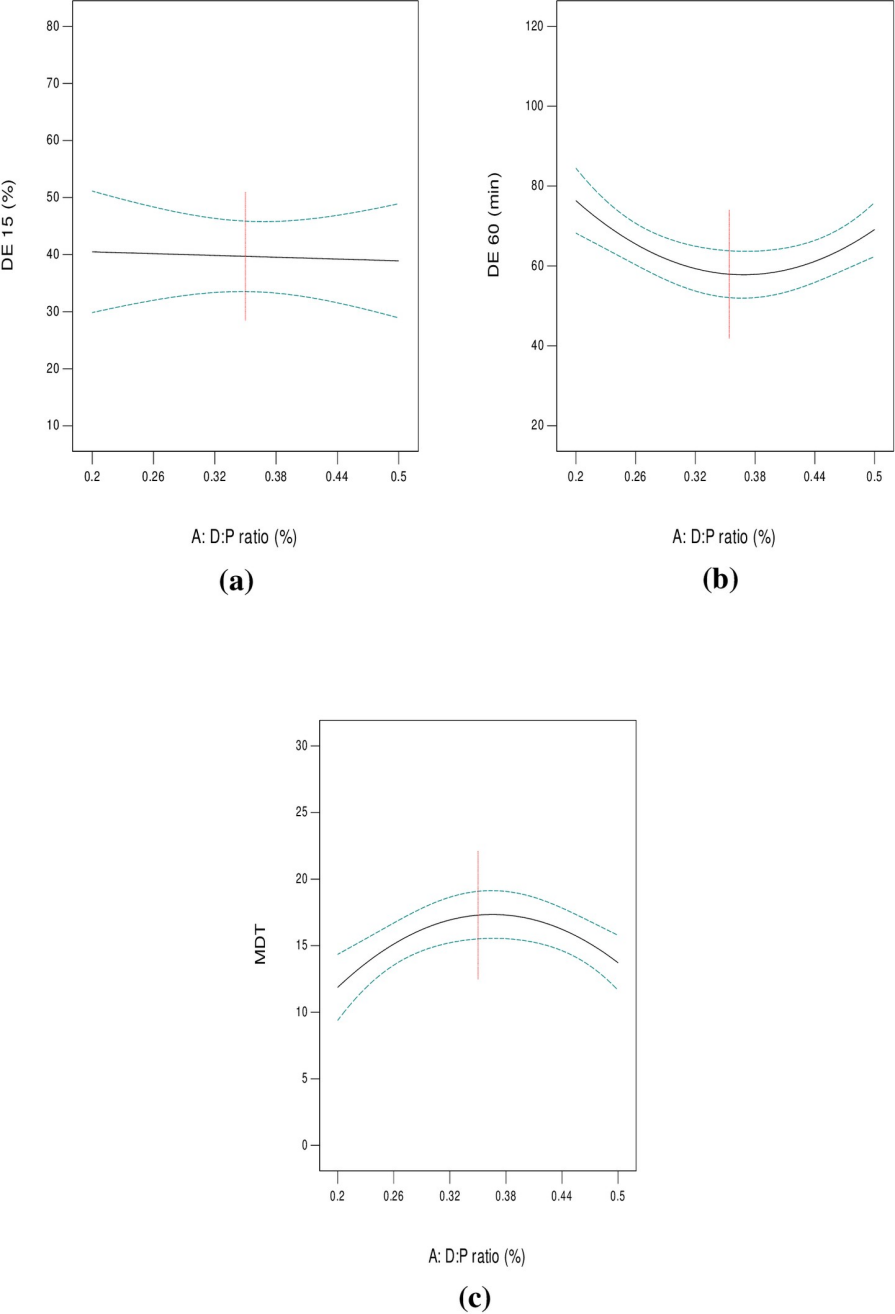
Line plots for the effect of D:P ratio on DE _(15 min)_ % (a), DE _(60 min)_ % (b) and MDT (c).

**Fig 10:**
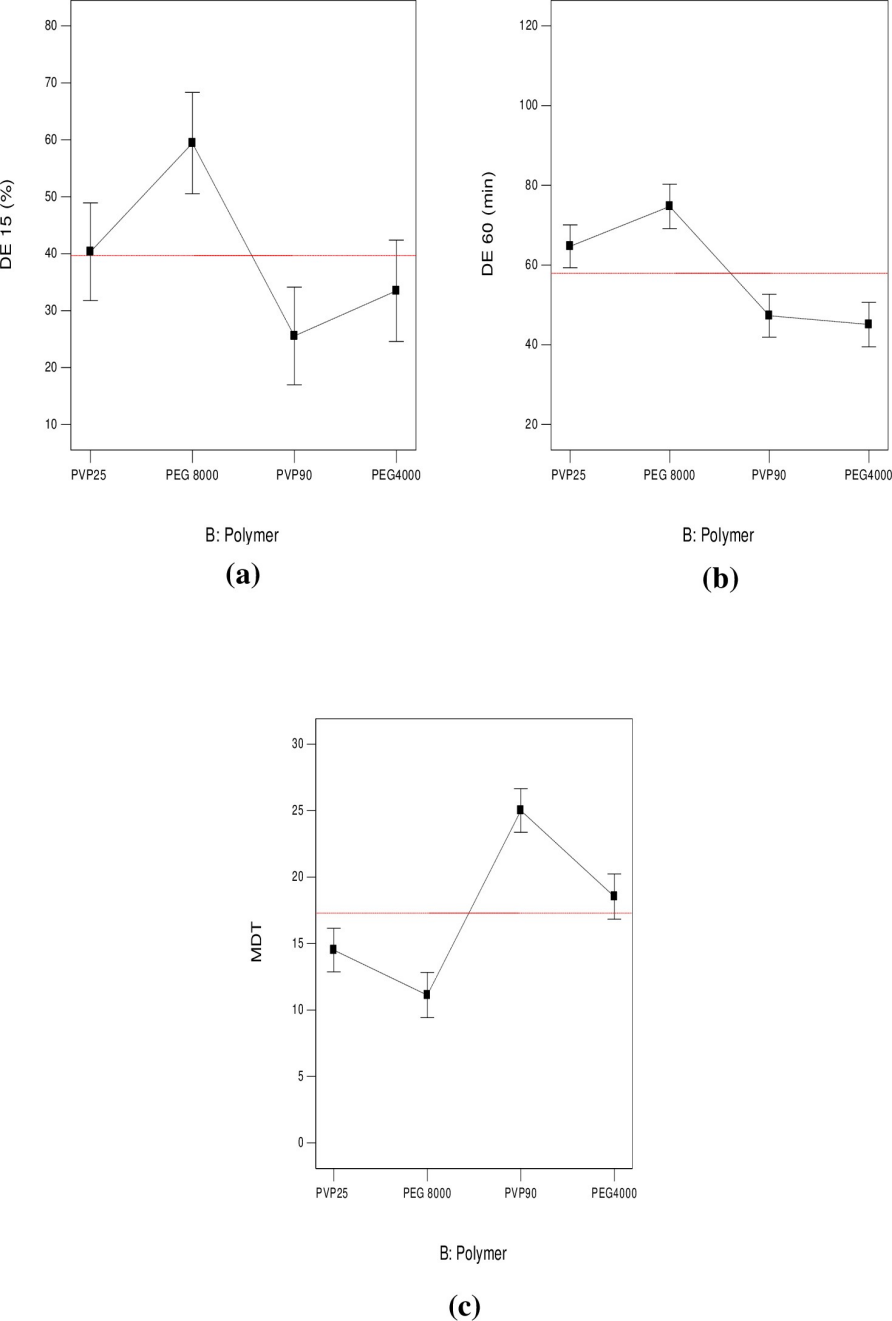
Line plots for the effect of polymer type on DE _(15 min)_ % (a), DE _(60 min)_ % (b) and MDT (c).

Results of calculated DE % at 15 and 60 min, as well as MDT are presented in Table 2. Among the prepared systems, SD8 exhibited the minimum DE _(15 min)_ % (13.69%) and DE _(60 min)_ % (40.03%), as well as the longest MDT (28.47 min). On the other hand, SD18 showed the maximum DE _(15 min)_ % (78.35%) and DE _(60 min)_ % (95.92%), as well as the shortest MDT (4.55 min). “Figs 9 and 10” show line plots for the effect of formulation variables on the measured dissolution parameters. ANOVA results revealed that both D:P ratio (A), as well as the type of the used polymer (B) significantly affected Y_2_, Y_3_ and Y_4_ (p < 0.0001). Similar results were obtained by Doshi et al. [47] in their study on carbamazepine. DCN dissolution rates from all SD were markedly higher than pure DCN in terms of DE _(15 min)_ % and DE _(60 min)_ % with values equal to 7.05% and 28.30% for plain drug, respectively. Although all the prepared systems showed enhanced DCN dissolution, yet their dissolution profiles were variable depending on both the implemented D:P ratio (A) and the type of the polymer used (B). As shown in Table 1, three levels of D:P ratio were studied and statistically analyzed. Results showed that SD containing equal D:P ratio (1:1 w/w) exhibited increased DE _(15 min)_ % and DE _(60 min)_ %. This can be explained by the fact that the fraction of drug per unit polymer within these systems exceeds the other ones and consequently, the polymer proportion included within them is the least among the prepared systems. This in return provided a passable way, that is less crowded, for the drug to diffuse easily into the dissolution medium, resulting in augmented drug dissolution. When D:P ratio increased to 1:2 (w/w), DE _(15 min)_ % and DE _(60 min)_ % significantly decreased (p < 0.0001), as shown in “Fig 9A and 9B”, respectively. This can be due to the increased thickness of the solid dispersions’ matrix that occurred upon increasing the polymer fraction, leading to slowing down drug diffusion into the dissolution medium. Similar findings were obtained by Kılıçarslan et al. [48] in their study on Verapamil HCl. However, DE _(15 min)_ % and DE _(60 min)_ % came back to increase upon increasing the D:P ratio to 1:4 w/w. These systems are characterized by low drug loading within readily soluble hydrophilic polymers. In this case, finely divided drug particles are closely surrounded by the hydrophilic polymer, preventing drug aggregation and allowing enhanced/faster dissolution. Similar findings were observed by [49–53]. In addition, high proportion of incorporated polymer, allowed more drug particles to become impregnated within their hydrophilic matrix, hence complete drug dispersion occurs. Similar findings were reported by Yang et al. [53] in their study on repaglinide and by Paradkar et al. [54] in their study on curcumin. From the previous results we can conclude that DCN dissolution exhibited a D:P ratio dependent behavior. MDT was also significantly affected by D:P ratio (p < 0.0001). Line plot for MDT (Fig 9C) shows an inverted superimposable diagram for DE % line plots. This is because they acquire an inversely proportional relationship where, increased DE % is associated with short MDT values and vice versa.

ANOVA results also revealed a significant effect of the type of polymers under study (B) on the measured responses (p < 0.0001) (Fig 10). Although all polymers under study acquire strong hydrophilic properties yet, their effect on DCN dissolution was variable. This can be attributed to difference in their molecular weights. Results showed that systems containing PEG 8000 achieved the highest DE _(15 min)_ % (Fig 10A) and DE _(60 min)_ % (Fig 10B), as well as the shortest MDT compared to PEG 4000 (Fig 10C). This can be due to higher hydrophilic properties acquired with high molecular weight PEGs. These findings were also reported by Bolourchian et al. [55]. Thus, the lower hydrophilicity of PEG 4000 compared to PEG 8000 can be the main reason for decreased DE % and longer MDT. Results also showed that SD prepared using PVP K25 acquired increased DE % and shorter MDT compared to systems containing PVP K90. A possible justification can be based on the fact that the higher the molecular weight of PVPs, the higher the viscosity of the diffusion layer surrounding drug particles and the lesser drug dissolution. Knopp et al. [46] achieved similar results in their study on indomethacin.

From the previous findings, it can be confirmed that enhanced dissolution rate of DCN is significantly affected by polymer proportion within the prepared SD, polymer hydrophilicity, as well as viscosity of dissolution medium surrounding the drug particles.

#### 3.3.3. Kinetic analysis

Zero order, first order and Higucchi diffusion kinetic models were employed to describe the mechanism of drug dissolution. Experimental data were fit to the three equations previously mentioned under section (2.5.3). Correlation coefficient (R^2^) values indicated the suitable kinetic model for drug dissolution mechanism. R^2^ values near to 1 denoted the best-fitted model.

In our study, first order and Higuchi diffusion model plots yielded greater R^2^ values than plots for zero order model (Table 4). Hence, dissolution profiles of plain DCN and SD (1–5), (8–11) as well as (15–18) followed the first order model. The rest the systems followed the Higuchi diffusion model. As reported by Wójcik et al. [56], dissolution of aqueous insoluble drugs from aqueous soluble systems is often described by the first order model kinetics. A possible justification for prevalence of the first order model may be because of the sooner dissolution of hydrophilic polymers, which results in formation of a boundary layer from which drug particles can dissolve. Also, this model describes that drug dissolution rate from the prepared SD is dependent on the proportion of incorporated hydrophilic polymers within the prepared systems. These results cohere very well with the results obtained from measured dissolution parameters. SD18 showed the highest dissolution rate constant (K = 0.449 min^-1^) within the first order model.

**Table 4:** Fitting results of dissolution profiles data of plain DCN and SD systems to distinguished models.

System	Correlation coefficient (R^2^)			Mechanism of DCN dissolution	K*
	Zero	First	Higuchi		
Plain DCN	0.9579	0.9832	0.8537	First order	0.012
SD1	0.0191	0.9533	0.7997	First order	0.072
SD2	0.0805	0.9533	0.8242	First order	0.064
SD3	-4.8566	0.4866	-0.3984	First order	0.107
SD4	-4.6977	0.1784	-0.3759	First order	0.095
SD5	0.6270	0.9890	0.9605	First order	0.050
SD6	0.5175	0.9001	0.9657	Higuchi	12.396
SD7	0.6294	0.9066	0.9576	Higuchi	12.073
SD8	0.9742	0.9753	0.8854	First order	0.020
SD9	0.9587	0.9857	0.9101	First order	0.021
SD10	0.0102	0.9740	0.9400	First order	0.062
SD11	-5.5721	-2.2280	-0.5790	First order	0.034
SD12	-1.1433	-0.1963	0.6864	Higuchi	8.482
SD13	-1.3041	-0.4011	0.6137	Higuchi	8.183
SD14	-0.4232	0.8472	0.8742	Higuchi	13.528
SD15	-102.4853	-6.0390	-37.1910	First order	0.250
SD16	-1.8328	0.9067	0.4140	First order	0.094
SD17	-1.5239	0.8918	0.5171	First order	0.086
SD18	-133.2485	0.7159	-48.0744	First order	0.449

*K = dissolution rate constant (min^-1^).

### 3.4. Formulation optimization

The suggested model for each of the measured responses was explored via ANOVA statistical analysis where, p value less than 0.5 indicated that the model is significant. Only DE _(15 min)_ % acquired a linear model while other responses followed a quadratic one (Table 5). The lack of fit was not significant for all models with p values equal to 0.4313, 0.2357, 0.7958 and 0.4764 for Y_1_, Y_2_, Y_3_ and Y_4_, respectively. High R^2^ values, displayed in Table 5, as well as good agreement between the adjusted R^2^ and the predicted R^2^ show that data were best fitted to the chosen models. In addition, high adequacy of the chosen models was confirmed by adequate precision values > 4 [57].

**Table 5:** Statistical parameters for selection of the best fit model of the measured responses.

Response	Model	p-value	Lack of fit (p-value)	R^2^	Adjusted R^2^	Predicted R^2^	Adequate precision	PRESS
Y_1_	Quadratic	< 0.0001	0.4313	0.9870	0.9755	0.9088	31.890	1.45
Y_2_	Linear	< 0.0001	0.2357	0.9999	0.999	0.9998	453.629	0.58
Y_3_	Linear	< 0.0001	0.7958	0.9953	0.9812	0.9523	666.062	0.43
Y_4_	Linear	< 0.0001	0.4764	0.999	0.999	0.996	446.789	0.23

**Fig 11:**
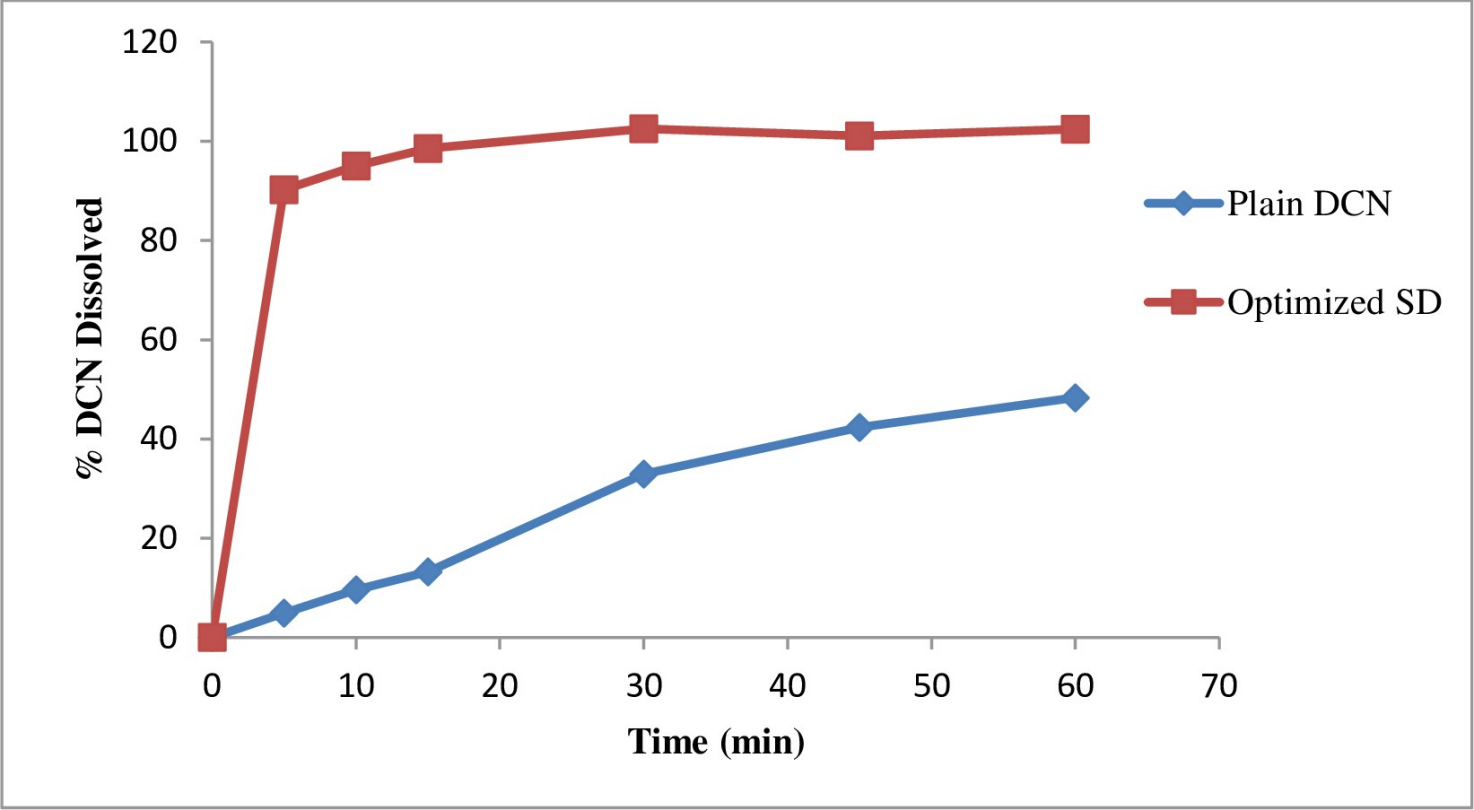
*In-vitro* dissolution profile of DCN from the optimized SD compared to plain DCN in phosphate buffer (pH 6.8).

In order to predict the values of formulation variables through which an optimized formulation can be achieved, formulation optimization was performed [58]. In our study, I-Optimal factorial design was chosen as it minimizes the average variance of prediction within the whole experimental region [59]. The experimental design consisted of 18 runs (Table 2). Effect of formulation variables on the measured responses was investigated via statistical analysis using Design Expert^®^ software. Based on desirability calculations, the chosen optimized system was SD18. It is composed of PEG 8000, having a D:P ratio of 1:4 w/w. Dissolution profiles of the optimized SD compared to plain drug is shown in “Fig 11”. Results showed that SD18 exhibited a 7.42 and 2.12 fold increase in DCN dissolution after 15 and 60 min, respectively compared to plain drug. Results also showed great similarity between observed and predicted values of the measured responses of the optimized system where it showed DC 99.06%, DE _(15 min)_ 76.35% and DE _(60 min)_ 96.89% and MDT of 4.10 min. Based on these results, the optimized system allowed 10.83 and 3.42 fold increase in DE _(15 min)_ %, DE _(60 min)_, respectively and 6.07 decrease in MDT compared to plain DCN. These results explain the powerful amorphization effect of PEG 8000 within the optimized SD. Incorporating PEG 8000 in a 1:4 ratio w/w augmented drug dispersal and increased polymer’s solubilizing efficiency. Similar results were obtained by Biswal et al. [52] in their study on PEG 8000 based solid dispersions.

### 3.5. Evaluation of optimized SD

#### 3.5.1. DSC

**Fig 12:**
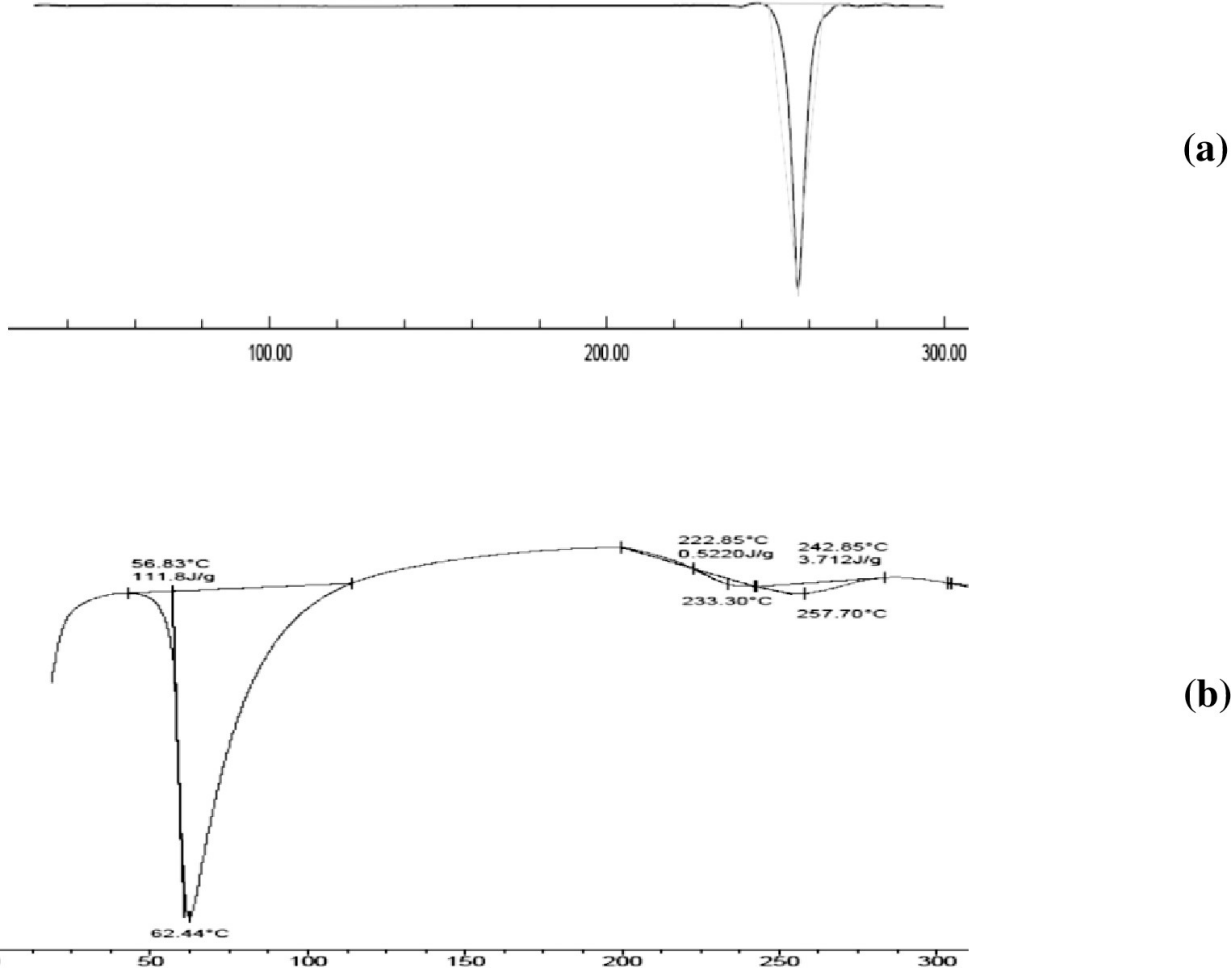
DSC thermograms of drug powder (a) and optimized SD (b).

Thermogram of the optimized SD presented nearly a flat line indicating absence of DCN endothermic peak (Fig 12), compared to plain drug. This could be noted due to the complete molecular dispersion of DCN within PEG 8000 which resulted in disappearance of drug crystallinity and its total amorphous transformation. This can be justified by drug-polymer interaction during the solvent evaporation process. This interaction occured via hydrogen bond formation resulted in entraping drug molecules within the hydrophilic polymer and inhibiting its crystallization. These results comply with previously reported enhanced *in*-*vitro* dissolution of DCN from the optimized system compared to plain drug (Fig 11). Similar results were obtained by [60, 52]. In order to confirm exclusion of crystallinity within the optimized system, XRD analysis was implemented.

#### 3.5.2. XRD

**Fig 13:**
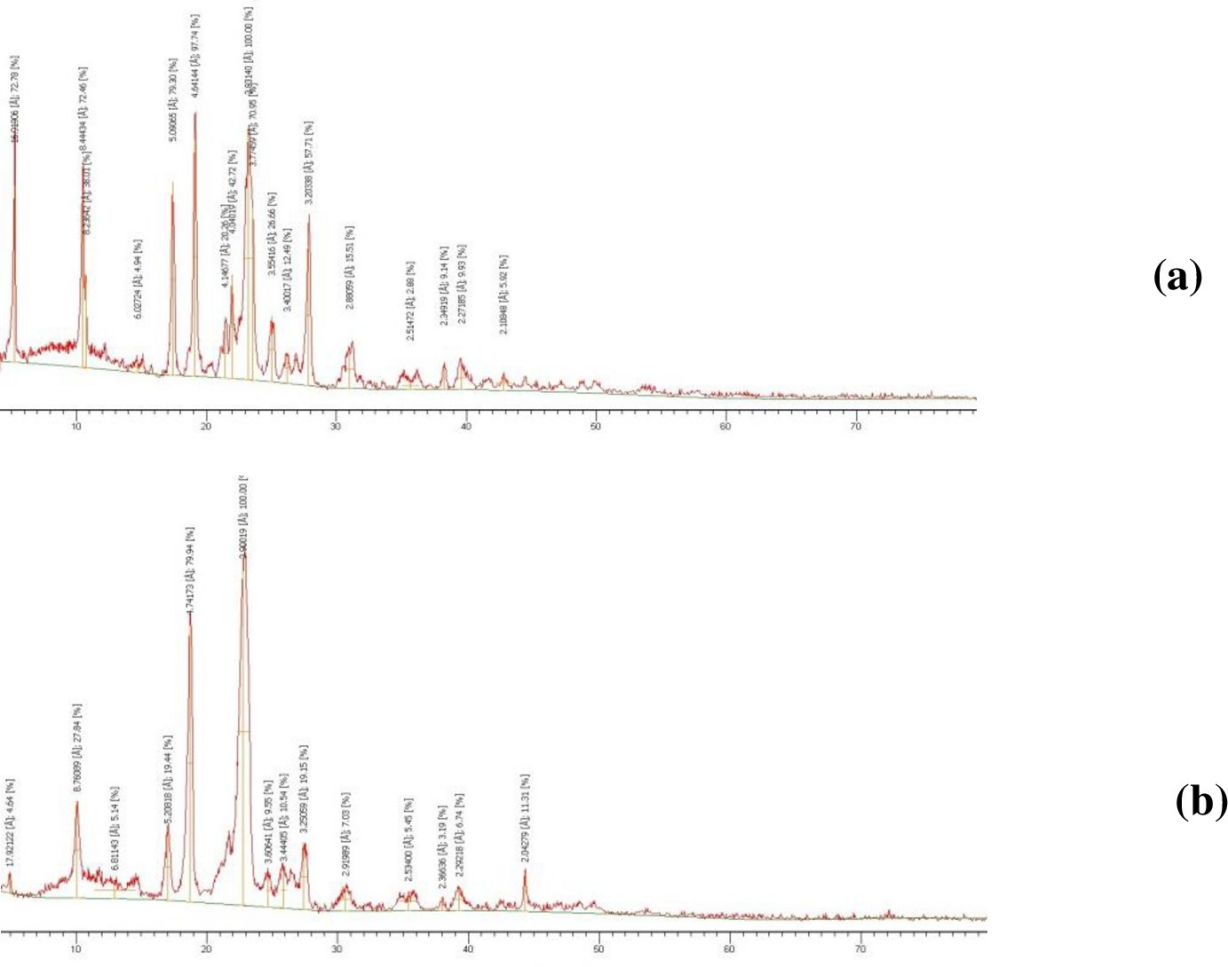
Powder X-ray diffraction spectra of drug powder (a) and optimized SD (b).

XRD analysis was performed for further investigation of the physical form of plain drug, as well as drug in the optimized system (Fig 13). The resulting diffractogram for plain drug (Fig 13A) showed three marked diffraction peaks with highest intensity at 2θ of 4°, 10.4° and 17.5° revealing its crystalline nature. Diffractogram of the optimized system (Fig 13B) showed prominent reduction and diminished intensity of DCN corresponding peaks reflecting its amorphous existence in the optimized system. This observed reduction in peak intensity is believed to be due to entrapment of drug within the incorporated polymer which again confirms DCN amorphization.

In order to compare the crystallinity in plain drug diffractogram with diffractogram of the optimized system, degree of relative crystallinity was calculated using the following formula:

$$DRC=I_{sample}/I_{plain\;drug}$$

Where, I_sample_ is the height intensity of the peak of the sample under investigation (optimized SD) and I_plain drug_ is the height intensity of the peak of plain drug at the same angle. The adopted peak was at 4°, where it was hard to distinguish its presence in the optimized system. The calculated DRC was 0.09. This value reflects the obviously reduced crystallinity of DCN within the optimized SD compared to plain drug. These results cohere with DSC results and again confirm the total dispersion of DCN in the hydrophilic polymer.

#### 3.5.3. SEM

**Fig 14:**
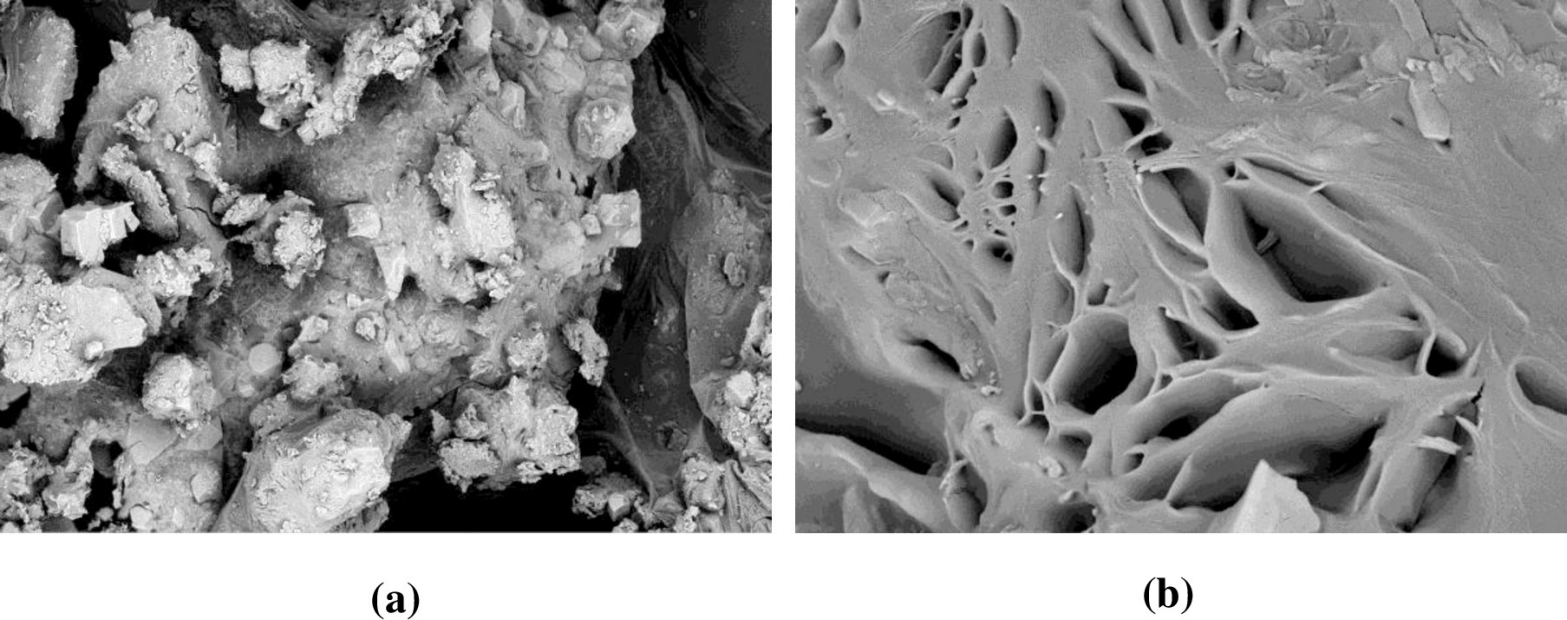
Scanning electron micrographs of pure DCN (a) and the optimized SD (b).

Surface views of scanning electron micrographs of pure DCN and the optimized SD are shown in “Fig 14”. Pure DCN micrograph (Fig 14A) showed large, irregular lumps of crystals. Disappearance of DCN characteristic lumps and appearance of a highly porous structure (Fig 14B) clearly confirms the amorphous transformation of the drug. Hence, formation of polymeric solid dispersions produced amorphous systems. These results verify the enhanced dissolution properties of DCN.

### 3.6. Physiologically-based pharmacokinetic modeling

Simcyp^®^-population based simulator software has been successfully used for modeling and simulation of ADME of APIs within various populations. As previously mentioned, it is based on combining the *in*-*vitro* experimental data resulting during pre-clinical studies and their relevant physicochemical parameters of the API under investigation together with physiological, demographic and genetic attributes of varying patients’ populations [61]. In our study, two types of populations were investigated namely; middle aged healthy adults and geriatrics. In order to predict the PK parameters of DCN in both populations following oral administration of the optimized SD, the investigated model should be priorly verified against reported clinical data. Model verification was performed by comparing PBPK model–simulated PK parameters (C_max_, T_max_ and AUC_0-24h_) of DCN following a single oral dose of 50 mg to correspondingly reported PK parameters [1] (Table 6). Results showed that the simulated/reported ratios for C_max_, T_max_ and AUC_0-24h_ were 0.99, 0.72 and 1.14, respectively for healthy adults and 1.04, 0.72 and 1.30, respectively for geriatrics. These findings confirm the validity of the investigated software and the adopted model. Hence, PBPK model can be employed to successfully predict the PK parameters of DCN.

**Table 6:** Comparison of PBPK-simulated model and reported DCN pharmacokinetic parameters following a single oral dose (50 mg) in healthy adults and geriatrics.

DCN treatment	^a^C_max_ (μg/mL)	^b^T_max_(h)	^a^ AUC_0-24h_ (μg/mL.h)
Simulated data of optimized SD in healthy adults	5.46 ± 0.97	1.80	35.53 ± 11.50
Simulated data of optimized SD in geriatrics	5.70 ± 0.96	1.80	40.56 ± 12.89
Simulated data of plain DCN	2.61 ± 0.61	1.85	15.48 ± 4.67
^c^Reported clinical data	5.47	2.50	31.11

^a^Mean

^b^Median

^c^Observed data as retrieved from [1].

**Fig 15:**
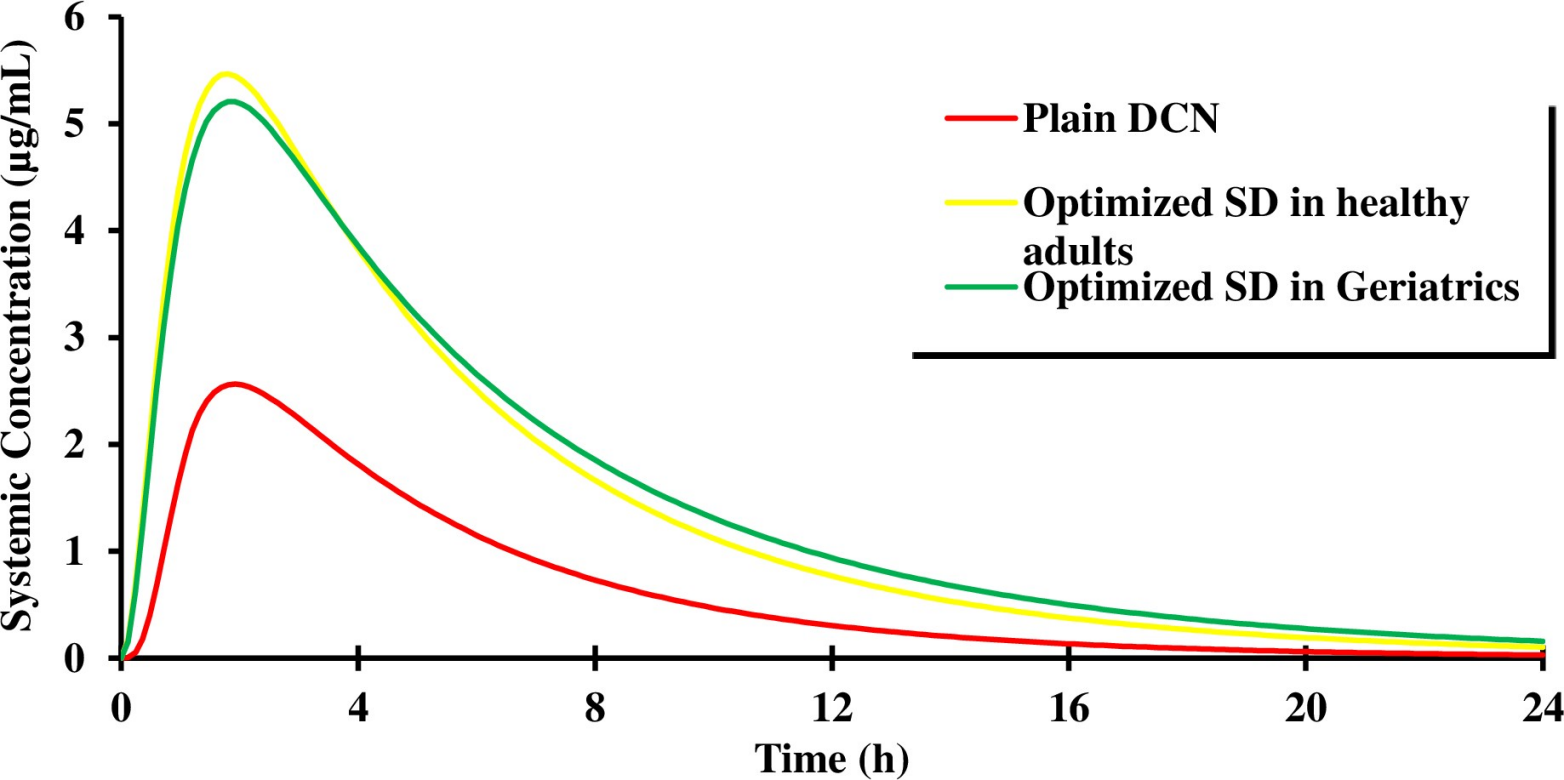
Physiologically based pharmacokinetic (PBPK) model–simulated DCN plasma concentration-time curves following oral administration of optimized SD and plain DCN in healthy adults and geriatrics.

“Fig 15” displays the PBPK model–simulated plasma concentration-time curves of DCN following oral administration of optimized SD in healthy adults (aged between 40 and 55) and geriatrics (aged between 65–75), relative to oral plain DCN. The predicted PK parameters are summarized in Table 6.

Results revealed that simulated data for both populations, healthy adults and geriatrics indicated that the optimized SD is superior to plain drug. The optimized SD exhibited a 2.1 and a 2.9 fold increase in C_max_ in healthy adults and geriatrics, respectively compared to plain drug yet, values were statistically insignificant (p = 0.1048 and 0.0865, respectively). Also, no significant difference was observed upon comparing medians of the T_max_ obtained from the optimized SD in both populations to that obtained from the plain drug (p > 0.05). However, statistically significant difference was found between AUC_0-24h_ determined for optimized SD in healthy adults (p = 0.003) and geriatrics (p = 0.0016), compared to plain drug. Based on the AUC_0-24h_ values in both populations, the relative bioavailability of the optimized SD compared to plain drug was found to be 229.52% and 262.02%, respectively. It is clear that bioavailability in geriatrics exceeded that in middle aged healthy adults. This is attributed to the significant physiological changes occurring with aging. These include reduced renal function due to decreased glomerular filtration rate. Similar results were obtained by Rhee et al. [62] in their PBPK modeling on metformin. Also, in geriatrics, liver mass, as well as hepatic perfusion are diminished leading to increased C_max_, AUC_0-24h_ and consequently bioavailability [63]. The enhanced oral bioavailability from the optimized SD compared to plain DCN is a reasonable consequence attributed to DCN amorphization and enhanced dissolution properties previously confirmed.

## 4. Conclusion

From this study, it is concluded that all solid dispersion systems prepared using different hydrophilic polymers, succeeded in enhancing DCN dissolution properties. The optimized SD, containing PEG 8000 (with a D:P ratio 1:4 w/w) showed the maximum enhancement in terms of the measured dissolution parameters, compared to pure drug. Results obtained from DSC, XRD and SEM analysis confirmed the conversion of DCN from crystalline to amorphous state which allied with the enhanced dissolution profile of the optimized SD. These results confirm the development of successful soluble complexes between DCN and the incorporated hydrophilic polymer. Therefore, the obtained results were further employed for simulation studies in an attempt to predict the enhanced bioavailability of the optimized SD compared to plain DCN. PBPK model of both middle aged healthy adults and geriatrics were successfully established using Simcyp^®^ software. The constructed models could adequately simulate PK parameters of DCN within the optimized SD. The developed optimized SD was found to exhibit significant increase in the AUC_0-24h_ and consequently augmented bioavailability, compared to plain drug in the selected populations. Amorphous formation and enhanced wettability of hydrophobic drug particles were the main reasons for dissolution and bioavailability enhancement of the optimized SD. This study can provide a preparatory example of implementing PBPK modeling to guide the development of BCS II APIs, as DCN prior to clinical trials.

## Supporting information

S1 File DSC compatibility studies. (XLSX)

S2 File Raw data for dissolution parameters of the prepared SD systems. (XLSX)

S3 File Statistical analysis—ANOVA results. (DOCX)

S4 File XRD data for plain DCN. (RTF)

S5 File XRD data for optimized SD. (RTF)

S6 File Simulation of DCN in healthy volunteers—PBPK modeling. (XLSX)

S7 File Simulation of optimized SD in geriatric patients—PBPK modeling. (XLSX)

S8 File Simulation of optimized SD in healthy volunteers—PBPK modeling. (XLSX)

S1 Graphical Abstract (PPTX)

## Abbreviations

DCN: Diacerein
OA: Osteoarthritis
NSAIDs: Non-steroidal anti-inflammatory drugs
BCS II: Biopharmaceutics Classification System, Class II
APIs: Active pharmaceutical ingredients
SD: Solid disersions
PVPs: Polyvinyl pyrrolidones
PEGs: Polyethylene glycols
D:P: Drug:Polymer
PK: Pharmacokinetic
IVIVC: *In*-*vitro*/*In*-*vivo* Correlation
PBPK: Physiologically based pharmacokinetic
ADME: Absorption, distribution, metabolism and excretion
BPA: Bisphenol A
PBTK: Physiologically Based Toxicokinetic
FDA: Food and Drug administration
PEG 4000: Polyethylene glycol 4000
PEG 8000: Polyethylene glycol 8000
PVP K25: Polyvinylpyrrolidone K25
PVP K90: Polyvinylpyrrolidone K90
DSC: Differential Scanning Calorimetry
PM: Physical mixtures
FTIR: Fourier transform infra-red spectroscopy
r.p.m: Revolutions per minute
*f*2: Similarity factor
DC: Drug content
DE %: Dissolution efficiency percent
MDT: Mean dissolution time
DE _(15 min)_ %: DE % at 15 min
DE _(60 min)_ %: DE % at 60 min
R^2^: Correlation coefficient
2FI: Two-factor interaction
R^2^: Multiple correlation coefficient
Adjusted R^2^: Adjusted multiple correlation coefficient
Predicted R^2^: Predicted multiple correlation coefficient
PRESS: Predicted residual sum of squares
ANOVA: Analysis of variance
XRD: X-ray diffraction
SEM analysis: Scanning electron microscopic analysis
ADAM: Advanced Dissolution, Absorption and Metabolism
C_max_: Maximum plasma concentration
T_max_: Time required to reach C_max_
AUC_0-24h_: Area under the plasma concentration time curve
h: Hour
min: Minutes
DRC: Degree of relative crystallinity
