# Supplementary material for: Preparation of solid dispersion systems for enhanced dissolution of poorly water soluble diacerein: In-vitro evaluation, optimization and physiologically based pharmacokinetic modeling
Source: PLoS One. 2021 Jan 20;16(1):e0245482. doi: 10.1371/journal.pone.0245482 (PMC7816977; doi:10.1371/journal.pone.0245482)
Supplement: S3 File — (DOCX) [file pone.0245482.s003.docx]

**Statistical analysis of the DCN-SD systems data**

1. **Statistical analysis for Drug content % response:**

**S1 Table: Sequential model sum of squares for DC %.**

| **Source** | **Sum of squares** | **DF*** | **Mean square** | **F value** | **p-value** | **Comments** |
| --- | --- | --- | --- | --- | --- | --- |
| Mean vs. Total | 164596.44 | 1 | 164596.44 |  |  |  |
| Linear vs. Mean | 6.34 | 4 | 1.58 | 2.15 | 0.1325 |  |
| 2FI vs. Linear | 8.20 | 3 | 2.73 | 19.73 | 0.0002 |  |
| **Quadratic vs. 2FI** | **1.18** | **1** | **1.18** | **51.31** | **< 0.0001** | **Suggested** |
| Cubic vs. Quadratic | 0.072 | 3 | 0.024 | 1.06 | 0.4313 | Aliased |
| Residual | 0.14 | 6 | 0.023 |  |  |  |
| Total | 164596.44 | 18 | 9145.13 |  |  |  |

*DF: Degrees of freedom

**S2 Table: Lack of fit of different models for DC %.**

| **Source** | **Sum of squares** | **DF*** | **Mean square** | **F value** | **p-value** | **Comments** |
| --- | --- | --- | --- | --- | --- | --- |
| Linear | 9.45 | 7 | 1.35 | 60.03 | < 0.0001 |  |
| 2FI | 1.25 | 4 | 0.31 | 13.90 | 0.0034 |  |
| **Quadratic** | **0.072** | **3** | **0.024** | **1.06** | **0.4313** | **Suggested** |
| Cubic | 0.000 | 0 |  |  |  | Aliased |
| Pure Error | 0.14 | 6 | 0.023 |  |  |  |

*DF: Degrees of freedom

**S3 Table: Model summary statistics for DC%.**

| **Source** | **SD*** | **R^2^** | **Adjusted R^2^** | **Prediction R^2^** | **PRESS** | **Comments** |
| --- | --- | --- | --- | --- | --- | --- |
| Linear | 0.86 | 0.3979 | 0.2127 | -0.2944 | 20.62 |  |
| 2FI | 0.37 | 0.9130 | 0.8520 | 0.4478 | 8.80 |  |
| **Quadratic** | **0.15** | **0.9870** | **0.9755** | **0.9088** | **1.45** | **Suggested** |
| Cubic | 0.15 | 0.9915 | 0.9760 |  | + | Aliased |

*SD: Standard deviation

**S4 Table: ANOVA table for the quadratic model for DC %.**

| **Source** | **Sum of squares** | **DF*** | **Mean square** | **F value** | **p-value** | **Comment** |
| --- | --- | --- | --- | --- | --- | --- |
| **Model** | **15.72** | **8** | **1.97** | **85.50** | **< 0.0001** | **significant** |
| *A-D:P ratio* | *0.24* | *1* | *0.24* | *10.39* | *0.0104* |  |
| *B-Polymer* | *6.22* | *3* | *2.07* | *90.13* | *< 0.0001* |  |
| *AB* | *7.71* | *3* | *2.57* | *111.77* | *< 0.0001* |  |
| *A^2^* | *1.18* | *1* | *1.18* | *51.31* | *< 0.0001* |  |
| Residual | 0.21 | 9 | 0.023 |  |  |  |
| ***Lack of Fit*** | ***0.072*** | ***3*** | ***0.024*** | ***1.06*** | ***0.4313*** | ***not significant*** |
| *Pure Error* | *0.14* | *6* | *0.023* |  |  |  |
| Cor Total | 15.93 | 17 |  |  |  |  |

DF: Degrees of freedom

1. **Statistical analysis for DE _(15 min)_ %:**

**S5 Table: Sequential model sum of squares for DE _(15 min)_ %.**

| **Source** | **Sum of squares** | **DF*** | **Mean square** | **F value** | **p-value** | **Comments** |
| --- | --- | --- | --- | --- | --- | --- |
| Mean vs. Total | 27244.78 | 1 | 27244.78 |  |  |  |
| **Linear vs. Mean** | **2758.14** | **4** | **689.53** | **30925.76** | **< 0.0001*** | **Suggested** |
| 2FI vs. Linear | 0.054 | 3 | 0.018 | 0.77 | 0.5385 |  |
| Quadratic vs. 2FI | 0.056 | 1 | 0.056 | 2.78 | 0.1299 |  |
| Cubic vs. Quadratic | 0.088 | 3 | 0.029 | 1.92 | 0.2270 | Aliased |
| Residual | 0.092 | 6 | 0.015 |  |  |  |
| Total | 30003.21 | 18 | 1666.85 |  |  |  |

* Significant

DF: Degrees of freedom

**S6 Table: Lack of fit of different models for DE _(15 min)_ %.**

| **Source** | **Sum of squares** | **DF** | **Mean square** | **F value** | **p-value** | **Comments** |
| --- | --- | --- | --- | --- | --- | --- |
| **Linear** | **0.20** | **7** | **0.028** | **1.85** | **0.2357** | **Suggested** |
| 2FI | 0.14 | 4 | 0.036 | 2.35 | 0.1673 |  |
| Quadratic | 0.088 | 3 | 0.029 | 1.92 | 0.2270 |  |
| Cubic | 0.000 | 0 |  |  |  | Aliased |
| Pure Error | 0.092 | 6 | 0.015 |  |  |  |

DF: Degrees of freedom

**S7 Table: Model summary statistics for DE _(15 min)_ %.**

| **Source** | **SD** | **R^2^** | **Adjusted R^2^** | **Prediction R^2^** | **PRESS** | **Comments** |
| --- | --- | --- | --- | --- | --- | --- |
| Linear | 0.15 | 0.9999 | 0.999 | 0.9998 | 0.58 | Suggested |
| 2FI | 0.15 | 0.985 | 0.982 | 0.9995 | 1.51 |  |
| Quadratic | 0.14 | 0.927 | 0.925 | 0.9995 | 1.31 |  |
| Cubic | 0.12 | 1.0000 | 0.9999 |  | + | Aliased |

SD: Standard deviation

**S8 Table: ANOVA table for the quadratic model for DE _(15 min)_ %.**

| **Source** | **Sum of squares** | **DF** | **Mean square** | **F value** | **p-value** | **Comment** |
| --- | --- | --- | --- | --- | --- | --- |
| Model | 2758.14 | 4 | 689.53 | 30925.7 | < 0.0001* | Significant |
| *A-D:P ratio* | *6.92* | *1* | *6.92* | *310.29* | *< 0.0001** |  |
| *B-Polymer* | *2713.62* | *3* | *904.54* | *40568.83* | *< 0.0001** |  |
| Residual | 0.29 | 13 | 0.022 |  |  |  |
| *Lack of Fit* | *0.20* | *7* | *0.028* | *1.85* | *0.2357* | *Not significant* |
| *Pure Error* | *0.092* | *6* | *0.015* |  |  |  |

* Significant

DF: Degrees of freedom

1. **Statistical analysis for DE _(60 min)_ %:**

**S9 Table: Sequential model sum of squares for DE _(60 min)_ %.**

| **Source** | **Sum of squares** | **DF** | **Mean square** | **F value** | **p-value** | **Comments** |
| --- | --- | --- | --- | --- | --- | --- |
| Mean vs. Total | 78072.95 | 1 | 78072.95 |  |  |  |
| Linear vs. Mean | 2597.23 | 4 | 649.31 | 8.35 | 0.0015 |  |
| 2FI vs. Linear | 174.87 | 3 | 58.29 | 0.70 | 0.5744 |  |
| **Quadratic vs. 2FI** | **835.33** | **1** | **835.33** | **73446.43** | **< 0.0001*** | **Suggested** |
| Cubic vs. Quadratic | 0.017 | 3 | 0.0056591 | 0.40 | 0.7598 | Aliased |
| Residual | 0.085 | 6 | 0.014 |  |  |  |
| Total | 81680.48 | 18 | 4537.80 |  |  |  |

* Significant

DF: Degrees of freedom

**S10 Table: Lack of fit of different models for DE _(60 min)_ %.**

| **Source** | **Sum of squares** | **DF** | **Mean square** | **F value** | **p-value** | **Comments** |
| --- | --- | --- | --- | --- | --- | --- |
| Linear | 1010.21 | 7 | 144.32 | 10141.38 | < 0.0001* |  |
| 2FI | 835.35 | 4 | 208.84 | 14675.38 | < 0.0001* |  |
| **Quadratic** | **0.017** | **3** | **0.0056591** | **0.40** | **0.7598** | **Suggested** |
| Cubic | 0.000 | 0 |  |  |  | Aliased |
| Pure Error | 0.085 | 6 | 0.014 |  |  |  |

* Significant

DF: Degrees of freedom

**S11 Table: Model summary statistics for DE _(60 min)_ %.**

| **Source** | **SD** | **R^2^** | **Adjusted R^2^** | **Prediction R^2^** | **PRESS** | **Comments** |
| --- | --- | --- | --- | --- | --- | --- |
| Linear | 8.82 | 0.7199 | 0.6338 | 0.4608 | 1945.24 |  |
| 2FI | 9.14 | 0.7684 | 0.6063 | -0.5253 | 5502.65 |  |
| **Quadratic** | **0.11** | **0.9953** | **0.9812** | **0.9523** | **0.43** | **Suggested** |
| Cubic | 0.12 | 1.0000 | 0.9999 |  | + | Aliased |

SD: Standard deviation

**S12 Table: ANOVA table for the quadratic model for DE _(60 min)_ %.**

| **Source** | **Sum of squares** | **DF** | **Mean square** | **F value** | **p-value** | **comment** |
| --- | --- | --- | --- | --- | --- | --- |
| Model | 3607.43 | 8 | 450.93 | 39647.94 | < 0.0001* | significant |
| *A-D:P ratio* | *28.26* | *1* | *28.26* | *2484.78* | *< 0.0001** |  |
| *B-Polymer* | *2589.07* | *3* | *863.02* | *75881.37* | *< 0.0001** |  |
| *AB* | *111.03* | *3* | *37.01* | *3254.24* | *< 0.0001** |  |
| *A^2^* | *835.33* | *1* | *835.33* | *73446.43* | *< 0.0001** |  |
| Residual | 0.10 | 9 | 0.011 |  |  |  |
| *Lack of Fit* | *0.017* | *3* | *0.00565* | *0.40* | *0.7598* | *not significant* |
| *Pure Error* | *0.085* | *6* | *0.014* |  |  |  |

* Significant

DF: Degrees of freedom

1. **Statistical analysis for MDT:**

**S13 Table: Sequential model sum of squares for MDT.**

| **Source** | **Sum of squares** | **DF** | **Mean square** | **F value** | **p-value** | **Comments** |
| --- | --- | --- | --- | --- | --- | --- |
| Mean vs. Total | 4089.09 | 1 | 4089.09 |  |  |  |
| Linear vs. Mean | 486.64 | 4 | 121.66 | 17.20 | < 0.0001* |  |
| 2FI vs. Linear | 7.12 | 3 | 2.37 | 0.28 | 0.8388 |  |
| **Quadratic vs. 2FI** | **84.82** | **1** | **84.82** | **20745.47** | **< 0.0001*** | **Suggested** |
| Cubic vs. Quadratic | 0.012 | 3 | 0.00393 | 0.94 | 0.4764 | Aliased |
| Residual | 0.025 | 6 | 0.00416 |  |  |  |
| Total | 4667.71 | 18 | 259.32 |  |  |  |

* Significant

DF: Degrees of freedom

**S14 Table: Lack of fit of different models for MDT.**

| **Source** | **Sum of squares** | **DF** | **Mean square** | **F value** | **p-value** | **Comments** |
| --- | --- | --- | --- | --- | --- | --- |
| Linear | 91.95 | 7 | 13.14 | 3152.64 | < 0.0001* |  |
| 2FI | 84.83 | 4 | 21.21 | 5089.77 | < 0.0001* |  |
| **Quadratic** | **0.012** | **3** | **0.00393** | **0.94** | **0.4764** | **Suggested** |
| Cubic | 0.000 | 0 |  |  |  | Aliased |
| Pure Error | 0.025 | 6 | 0.00416 |  |  |  |

* Significant

DF: Degrees of freedom

**S15 Table: Model summary statistics for MDT.**

| **Source** | **SD** | **R^2^** | **Adjusted R^2^** | **Prediction R^2^** | **PRESS** | **Comments** |
| --- | --- | --- | --- | --- | --- | --- |
| Linear | 2.66 | 0.8410 | 0.7921 | 0.6832 | 183.29 |  |
| 2FI | 2.91 | 0.8533 | 0.7507 | 0.0332 | 559.41 |  |
| **Quadratic** | **0.064** | **0.9999** | **0.9999** | **0.9996** | **0.23** | **Suggested** |
| Cubic | 0.065 | 1.0000 | 0.9999 |  | + | Aliased |

SD: Standard deviation

**S16 Table: ANOVA table for the quadratic model for MDT.**

| **Source** | **Sum of squares** | **DF** | **Mean square** | **F value** | **p-value** | **comment** |
| --- | --- | --- | --- | --- | --- | --- |
| Model | 578.58 | 8 | 72.32 | 17689.26 | < 0.0001* | significant |
| *A-D:P ratio* | *1.52* | *1* | *1.52* | *370.93* | *< 0.0001** |  |
| *B-Polymer* | *492.66* | *3* | *164.22* | *40166.62* | *< 0.0001** |  |
| *AB* | *8.00* | *3* | *2.67* | *652.33* | *< 0.0001** |  |
| *A^2^* | *84.82* | *1* | *84.82* | *20745.47* | *< 0.0001** |  |
| Residual | 0.037 | 9 | 0.004088 |  |  |  |
| *Lack of Fit* | *0.012* | *3* | *0.0039* | *0.94* | *0.4764* | *not significant* |
