# Supplementary material for: Preparation of solid dispersion systems for enhanced dissolution of poorly water soluble diacerein: In-vitro evaluation, optimization and physiologically based pharmacokinetic modeling
Source: PLoS One. 2021 Jan 20;16(1):e0245482. doi: 10.1371/journal.pone.0245482 (PMC7816977; doi:10.1371/journal.pone.0245482)
Supplement: S4 File — (RTF) [file pone.0245482.s004.RTF]

Anchor Scan Parameters

Dataset Name:	1
File name:	C:\XRD Data\New Services\Fady Adel\1.xrdml
Comment:	Configuration=Flat Sample Stage, Owner=User-1, Creation date=2/9/2011 11:00:54 AM
	Goniometer=Theta/Theta; Minimum step size 2Theta:0.0001; Minimum step size Omega:0.0001
	Sample stage=Stage for flat samples/holders
	Diffractometer system=EMPYREAN
	Measurement program=Aisha, Owner=User-1, Creation date=6/20/2011 11:07:36 AM
Measurement Date / Time:	1/20/2019 10:38:15 AM
Operator:	TEST1
Raw Data Origin:	XRD measurement (*.XRDML)
Scan Axis:	Gonio
Start Position [°2Th.]:	4.0150
End Position [°2Th.]:	79.9610
Step Size [°2Th.]:	0.0260
Scan Step Time [s]:	18.8700
Scan Type:	Continuous
PSD Mode:	Scanning
PSD Length [°2Th.]:	3.35
Offset [°2Th.]:	0.0000
Divergence Slit Type:	Fixed
Divergence Slit Size [°]:	0.0573
Specimen Length [mm]:	10.00
Measurement Temperature [°C]:	25.00
Anode Material:	Cu
K-Alpha1 [Å]:	1.54060
K-Alpha2 [Å]:	1.54443
K-Beta [Å]:	1.39225
K-A2 / K-A1 Ratio:	0.50000
Generator Settings:	30 mA, 45 kV
Diffractometer Type:	0000000011089631
Diffractometer Number:	0
Goniometer Radius [mm]:	240.00
Dist. Focus-Diverg. Slit [mm]:	100.00
Incident Beam Monochromator:	No
Spinning:	No


Graphics

     


Peak List

Pos.[°2Th.]  Height [cts]  FWHMLeft[°2Th.]  d-spacing [Å]  Rel. Int. [%]
   5.219(2)       804(19)         0.173(4)       16.91906          72.78  
  10.468(3)       800(14)         0.326(7)        8.44434          72.46  
  10.733(4)       420(21)          0.08(1)        8.23642          38.01  
   14.69(2)         55(3)          0.63(4)        6.02724           4.94  
  17.406(2)       876(11)         0.268(5)        5.09065          79.30  
  19.106(2)      1080(16)         0.252(5)        4.64144          97.74  
   21.41(1)       224(10)          0.45(3)        4.14677          20.26  
  21.983(5)       472(10)          0.32(1)        4.04019          42.72  
  23.197(6)      1104(12)          0.64(1)        3.83140         100.00  
  23.551(7)       784(26)          0.13(1)        3.77459          70.95  
  25.034(7)        294(8)          0.40(2)        3.55416          26.66  
  26.188(9)        138(4)          0.44(3)        3.40017          12.49  
  27.828(2)       637(10)         0.343(7)        3.20338          57.71  
  31.020(9)        171(7)          0.72(2)        2.88059          15.51  
   35.67(4)         32(6)          1.30(9)        2.51472           2.88  
  38.283(3)        101(8)         0.083(7)        2.34919           9.14  
   39.64(1)        110(4)          0.78(3)        2.27185           9.93  
   42.86(3)         65(2)          1.24(7)        2.10848           5.92  


Document History

Insert Measurement:
- File name = "1.xrdml"
- Modification time = "1/20/2019 1:22:17 PM"
- Modification editor = "TEST1"

Default properties:
- Measurement step axis = "None"
- Internal wavelengths used from anode material: Copper (Cu)
- Original K-Alpha1 wavelength = "1.54060"
- Used K-Alpha1 wavelength = "1.54060"
- Original K-Alpha2 wavelength = "1.54443"
- Used K-Alpha2 wavelength = "1.54443"
- Original K-Beta wavelength = "1.39225"
- Used K-Beta wavelength = "1.39225"
- Irradiated length = "10.00000"
- Spinner used = "No"
- Receiving slit size = "0.10000"
- Distance to sample = "Diffracted radius"
- Step axis value = "0.00000"
- Offset = "0.00000"
- Sample length = "10.00000"
- Modification time = "1/20/2019 1:22:17 PM"
- Modification editor = "TEST1"

Interpolate Step Size:
- Derived = "Yes"
- Step Size = "0.01"
- Modification time = "1/20/2019 1:22:17 PM"
- Modification editor = "PANalytical"

Determine Background:
- Add to net scan = "Nothing"
- User defined intensity = "-5"
- Correction method = "Automatic"
- Bending factor = "0"
- Minimum significance = "0.7"
- Minimum tip width = "0"
- Maximum tip width = "1"
- Peak base width = "2"
- Use smoothed input data = "No"
- Granularity = "25"
- Modification time = "5/21/2018 12:13:45 PM"
- Modification editor = "TEST1"

Edit Method:
- Old Value  = "Polynomial"
- Modification time = "1/20/2019 1:22:29 PM"
- Modification editor = "TEST1"

Search Peaks:
- Minimum significance = "3"
- Minimum tip width = "0.05"
- Maximum tip width = "1"
- Peak base width = "2"
- Method = "Minimum 2nd derivative"
- Modification time = "3/23/2015 1:21:51 PM"
- Modification editor = "TEST1"

Profile fitting:
- Angular range [°2Th.] = "4.015 - 79.961"
- Step No. 1
- Title = "Flat Background"
- Min. Shift/ESD = "0.1"
- Switch off after usage = "False"
- Step No. 2
- Title = "More background"
- Min. Shift/ESD = "0.1"
- Switch off after usage = "False"
- No. additional parameters = "3"
- Use 1/X background too = "True"
- Step No. 3
- Title = "Peak Position"
- Min. Shift/ESD = "0.1"
- Switch off after usage = "False"
- Step No. 4
- Title = "Peak Height"
- Min. Shift/ESD = "0.1"
- Switch off after usage = "False"
- Step No. 5
- Title = "Peak FWHM"
- Min. Shift/ESD = "0.1"
- Switch off after usage = "False"
- Step No. 6
- Title = "Peak Shape"
- Min. Shift/ESD = "0.1"
- Switch off after usage = "False"
- No. of refined parameters = "72"
- Chi Square = "2.27265011123179"
- Rp = "0.14499"
- Rwp = "0.17816"
- Rexp = "0.07839"
- Modification time = "1/20/2019 1:22:42 PM"
- Modification editor = "TEST1"

Delete All K-Alpha2 Peaks:
- Modification time = "1/20/2019 1:22:47 PM"
- Modification editor = "TEST1"

Smooth:
- Polynomial type = "Low pass"
- Convolution range = "11"
- Degree of smoothing = "1"
- Fast Fourier = "No"
- Omit Peaks = "Yes"
- Modification time = "4/19/2018 10:29:19 AM"
- Modification editor = "TEST1"
