# Supplementary material for: Preparation of solid dispersion systems for enhanced dissolution of poorly water soluble diacerein: In-vitro evaluation, optimization and physiologically based pharmacokinetic modeling
Source: PLoS One. 2021 Jan 20;16(1):e0245482. doi: 10.1371/journal.pone.0245482 (PMC7816977; doi:10.1371/journal.pone.0245482)
Supplement: S5 File — (RTF) [file pone.0245482.s005.RTF]

Anchor Scan Parameters

Dataset Name:	2
File name:	C:\XRD Data\New Services\Fady Adel\2.xrdml
Comment:	Configuration=Flat Sample Stage, Owner=User-1, Creation date=2/9/2011 11:00:54 AM
	Goniometer=Theta/Theta; Minimum step size 2Theta:0.0001; Minimum step size Omega:0.0001
	Sample stage=Stage for flat samples/holders
	Diffractometer system=EMPYREAN
	Measurement program=Aisha, Owner=User-1, Creation date=6/20/2011 11:07:36 AM
Measurement Date / Time:	1/20/2019 10:44:01 AM
Operator:	TEST1
Raw Data Origin:	XRD measurement (*.XRDML)
Scan Axis:	Gonio
Start Position [°2Th.]:	4.0150
End Position [°2Th.]:	79.9610
Step Size [°2Th.]:	0.0260
Scan Step Time [s]:	18.8700
Scan Type:	Continuous
PSD Mode:	Scanning
PSD Length [°2Th.]:	3.35
Offset [°2Th.]:	0.0000
Divergence Slit Type:	Fixed
Divergence Slit Size [°]:	0.0573
Specimen Length [mm]:	10.00
Measurement Temperature [°C]:	25.00
Anode Material:	Cu
K-Alpha1 [Å]:	1.54060
K-Alpha2 [Å]:	1.54443
K-Beta [Å]:	1.39225
K-A2 / K-A1 Ratio:	0.50000
Generator Settings:	30 mA, 45 kV
Diffractometer Type:	0000000011089631
Diffractometer Number:	0
Goniometer Radius [mm]:	240.00
Dist. Focus-Diverg. Slit [mm]:	100.00
Incident Beam Monochromator:	No
Spinning:	No


Graphics

     


Peak List

Pos.[°2Th.]  Height [cts]  FWHMLeft[°2Th.]  d-spacing [Å]  Rel. Int. [%]
    4.93(1)         69(6)          0.24(3)       17.92122           4.64  
  10.088(5)        415(6)          0.64(1)        8.76089          27.84  
   12.99(5)         77(3)           3.2(1)        6.81143           5.14  
  17.011(5)        290(6)          0.41(1)        5.20818          19.44  
  18.698(3)      1191(21)         0.380(8)        4.74173          79.94  
  22.782(5)      1489(12)          0.89(1)        3.90019         100.00  
   24.67(1)        142(5)          0.37(4)        3.60641           9.55  
  25.848(7)        157(5)          0.49(2)        3.44405          10.54  
  27.416(9)        285(8)          0.53(2)        3.25059          19.15  
   30.59(1)        105(3)          0.89(4)        2.91989           7.03  
   35.39(3)         81(4)          1.33(7)        2.53400           5.45  
  37.994(8)         47(4)          0.11(1)        2.36636           3.19  
   39.27(1)        100(3)          0.79(4)        2.29218           6.74  
  44.306(6)        169(7)          0.30(2)        2.04279          11.31  


Document History

Insert Measurement:
- File name = "2.xrdml"
- Modification time = "1/20/2019 1:23:43 PM"
- Modification editor = "TEST1"

Default properties:
- Measurement step axis = "None"
- Internal wavelengths used from anode material: Copper (Cu)
- Original K-Alpha1 wavelength = "1.54060"
- Used K-Alpha1 wavelength = "1.54060"
- Original K-Alpha2 wavelength = "1.54443"
- Used K-Alpha2 wavelength = "1.54443"
- Original K-Beta wavelength = "1.39225"
- Used K-Beta wavelength = "1.39225"
- Irradiated length = "10.00000"
- Spinner used = "No"
- Receiving slit size = "0.10000"
- Distance to sample = "Diffracted radius"
- Step axis value = "0.00000"
- Offset = "0.00000"
- Sample length = "10.00000"
- Modification time = "1/20/2019 1:23:43 PM"
- Modification editor = "TEST1"

Interpolate Step Size:
- Derived = "Yes"
- Step Size = "0.01"
- Modification time = "1/20/2019 1:23:43 PM"
- Modification editor = "PANalytical"

Determine Background:
- Correction method = "Manual"
- Use cubic spline interpolation = "No"
- Background base points = "4.01503028 172,6.87503027999998 143,15.8970302799999 134.863028377553,29.1570302799998 84,41.4810302799997 88,51.5950302799996 71,62.2810302799996 46,73.9810302799995 34.3932987202129,79.9610302799994 52.169416976374"
- Modification time = "1/20/2019 1:24:02 PM"
- Modification editor = "TEST1"

Edit Method:
- Old Value  = "Polynomial"
- Modification time = "1/20/2019 1:24:10 PM"
- Modification editor = "TEST1"

Search Peaks:
- Minimum significance = "3"
- Minimum tip width = "0.05"
- Maximum tip width = "1"
- Peak base width = "2"
- Method = "Minimum 2nd derivative"
- Modification time = "3/23/2015 1:21:51 PM"
- Modification editor = "TEST1"

Profile fitting:
- Angular range [°2Th.] = "4.015 - 79.961"
- Step No. 1
- Title = "Flat Background"
- Min. Shift/ESD = "0.1"
- Switch off after usage = "False"
- Step No. 2
- Title = "More background"
- Min. Shift/ESD = "0.1"
- Switch off after usage = "False"
- No. additional parameters = "3"
- Use 1/X background too = "True"
- Step No. 3
- Title = "Peak Position"
- Min. Shift/ESD = "0.1"
- Switch off after usage = "False"
- Step No. 4
- Title = "Peak Height"
- Min. Shift/ESD = "0.1"
- Switch off after usage = "False"
- Step No. 5
- Title = "Peak FWHM"
- Min. Shift/ESD = "0.1"
- Switch off after usage = "False"
- Step No. 6
- Title = "Peak Shape"
- Min. Shift/ESD = "0.1"
- Switch off after usage = "False"
- No. of refined parameters = "56"
- Chi Square = "2.03706928083115"
- Rp = "0.13031"
- Rwp = "0.16704"
- Rexp = "0.08200"
- Modification time = "1/20/2019 1:24:25 PM"
- Modification editor = "TEST1"

Delete All K-Alpha2 Peaks:
- Modification time = "1/20/2019 1:24:35 PM"
- Modification editor = "TEST1"

Smooth:
- Polynomial type = "Low pass"
- Convolution range = "11"
- Degree of smoothing = "1"
- Fast Fourier = "No"
- Omit Peaks = "Yes"
- Modification time = "4/19/2018 10:29:19 AM"
- Modification editor = "TEST1"
