# Supplementary material for: Preparation of solid dispersion systems for enhanced dissolution of poorly water soluble diacerein: In-vitro evaluation, optimization and physiologically based pharmacokinetic modeling
Source: PLoS One. 2021 Jan 20;16(1):e0245482. doi: 10.1371/journal.pone.0245482 (PMC7816977; doi:10.1371/journal.pone.0245482)
Supplement: S1 Graphical Abstract — (PPTX) [file pone.0245482.s009.pptx]

## Slide 1
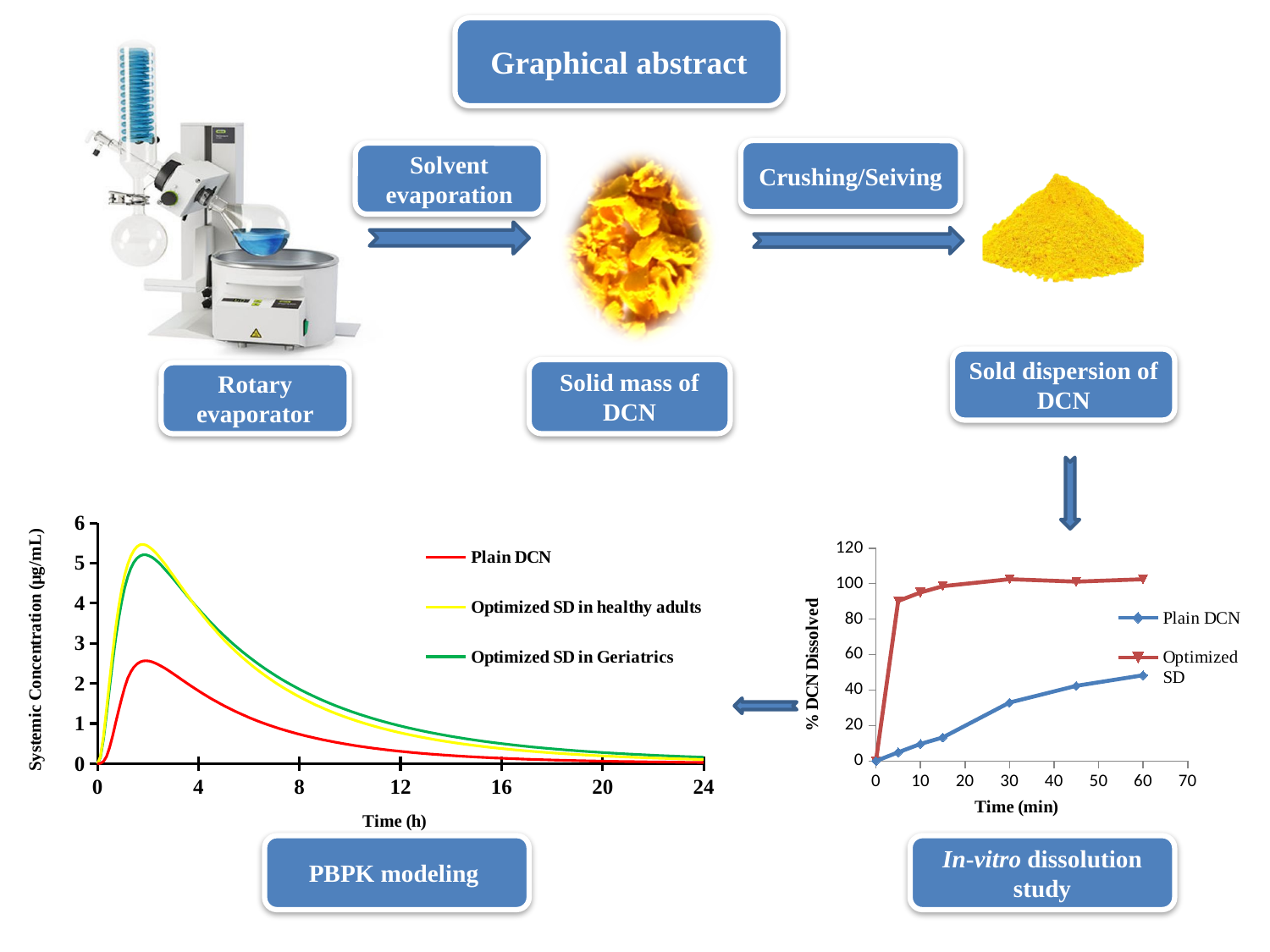

Graphical abstract
Crushing/Seiving
Solvent evaporation
Sold dispersion of DCN
Solid mass of DCN
Rotary evaporator
### Chart
| Category | | | |
|---|---|---|---|
### Chart
| Category | Plain DCN | Optimized SD |
|---|---|---|
PBPK modeling
In-vitro dissolution study
